# Cell Therapy: Types, Regulation, and Clinical Benefits

**DOI:** 10.3389/fmed.2021.756029

**Published:** 2021-11-22

**Authors:** Abed El-Hakim El-Kadiry, Moutih Rafei, Riam Shammaa

**Affiliations:** ^1^Laboratory of Thrombosis and Hemostasis, Montreal Heart Institute, Research Center, Montreal, QC, Canada; ^2^Department of Biomedical Sciences, Université de Montréal, Montreal, QC, Canada; ^3^Department of Pharmacology and Physiology, Université de Montréal, Montreal, QC, Canada; ^4^Department of Microbiology, Infectious Diseases and Immunology, Université de Montréal, Montreal, QC, Canada; ^5^Molecular Biology Program, Université de Montréal, Montreal, QC, Canada; ^6^Department of Microbiology and Immunology, McGill University, Montreal, QC, Canada; ^7^Canadian Centre for Regenerative Therapy, Toronto, ON, Canada; ^8^Department of Family and Community Medicine, University of Toronto, Toronto, ON, Canada

**Keywords:** cell therapy, FDA regulations, multicellular therapies, regenerative medicine, cancer, immune diseases, bone marrow aspirate concentrate (BMAC), mesenchymal stem cells

## Abstract

Cell therapy practices date back to the 19^th^ century and continue to expand on investigational and investment grounds. Cell therapy includes stem cell- and non–stem cell-based, unicellular and multicellular therapies, with different immunophenotypic profiles, isolation techniques, mechanisms of action, and regulatory levels. Following the steps of their predecessor cell therapies that have become established or commercialized, investigational and premarket approval-exempt cell therapies continue to provide patients with promising therapeutic benefits in different disease areas. In this review article, we delineate the vast types of cell therapy, including stem cell-based and non–stem cell-based cell therapies, and create the first-in-literature compilation of the different “multicellular” therapies used in clinical settings. Besides providing the nuts and bolts of FDA policies regulating their use, we discuss the benefits of cell therapies reported in 3 therapeutic areas—regenerative medicine, immune diseases, and cancer. Finally, we contemplate the recent attention shift toward combined therapy approaches, highlighting the factors that render multicellular therapies a more attractive option than their unicellular counterparts.

## Introduction

Cell therapy refers to the transfer of autologous or allogeneic cellular material into a patient for medical purposes (1, 2). The year 1889 witnessed the first practices of cell therapy by Charles-Édouard Brown-Séquard—pioneer in hormone therapy at the time—who attempted to suppress the effects of aging using injections of animal testicle extracts (3). Today, cell therapy continues to evolve with ongoing investigations for clinical safety and efficacy, and with a global market size estimated to expand from USD 9.5 billion in 2021 to USD 23.0 billion in 2028 (4). Cell therapy combines stem cell- and non–stem cell-based unicellular or multicellular therapies. It typically employs autologous or allogeneic cells; might involve genetic engineering or manipulations in formulation; and can be administered topically or as injectables, infusions, bioscaffolds, or scaffold-free systems (5–9). Cell therapy spans multiple therapeutic areas, such as regenerative medicine, immunotherapy, and cancer therapy. Currently, most cell therapies are in early stages of development (phase 1/2), with several exceptions being either a current best practice in specific settings (e.g., bone marrow/stem cell transplants, hepatocyte transplantation, skin equivalents), or approved for specific indications, such as PROVENGE^®^ (sipuleucel-T), LAVIV^®^ (azficel-T), MACI^®^ (autologous cultured chondrocytes on porcine collagen), and KYMRIAH™ (tisagenlecleucel) among others (Table 1) (5, 9, 27). Herein, we describe the different types of cell therapies, including stem cell-based and non–stem cell-based cell therapies, providing an overview of their nature as well as isolation and characterization techniques. We further create the first-in-literature portfolio for the different “multicellular” therapies, delineating their different cellular components and areas of use. In addition to reviewing the FDA's regulatory guidelines governing their use, we dive into the pros of cell therapies reported in regenerative medicine, immune system disorders, and cancer. Finally, we give our perspective on why multicellular therapies could contribute to more beneficial clinical outcomes compared to unicellular therapies, and how their development could be optimized for faster commercialization.

**Table 1:** FDA-approved cell therapy products.

**Therapy**	**Description**	**Indication**	**Mechanism of action**	**Initial US approval date**	**Manufacturer**	**References**
ALLOCORD (HPC, Cord Blood)	Consists of allogeneic cord blood hematopoietic progenitor cells (HPCs), monocytes, lymphocytes, and granulocytes	Hematopoietic system disorders	Hematopoietic stem/progenitor cells from HPC, Cord Blood migrate to the bone marrow where they divide and mature; mature cells are then released into the blood, restoring counts and functions of bone marrow cells. Mature leukocytes may also synthesize enzymes that might improve cellular functions of host tissues (mechanism of action unknown)	2013	SSM Cardinal Glennon Children's Medical Center	(10)
CLEVECORD (HPC, Cord Blood)				2016	Cleveland Cord Blood Center	(11)
HEMACORD (HPC, Cord Blood)				2011	New York Blood Center, Inc.	(12)
DUCORD (HPC, Cord Blood)				2012	Duke University School of Medicine	(13)
HPC, Cord Blood				2012	ClinImmune Labs	(14)
HPC, Cord Blood				2018	MD Anderson Cord Blood Bank	(15)
HPC, Cord Blood				2013	LifeSouth Community Blood Centers, Inc.	(16)
HPC, Cord Blood				2016	Bloodworks	(17)
BREYANZI^®^ (lisocabtagene maraleucel)	CD19-directed genetically modified autologous T cell immunotherapy	Relapsed or refractory large B-cell lymphom	CAR binding to CD19 expressed on tumor and normal B cells induces activation and proliferation of CAR T cells and cytotoxic killing of target cells	2021	Juno Therapeutics, Inc., a Bristol-Myers Squibb Company	(18)
KYMRIAH™ (tisagenlecleucel)		Relapsed or refractory B-cell precursor acute lymphoblastic leukemia		2017	Novartis Pharmaceuticals Corporation	(19)
		Relapsed or refractory large B-cell lymphoma				
YESCARTA^®^ (axicabtagene ciloleucel)		Relapsed or refractory large B-cell lymphoma		2017	Kite Pharma, Inc.	(20)
TECARTUS™ (brexucabtagene autoleucel)		Relapsed or refractory mantle cell lymphoma		2020		(21)
ABECMA^®^ (idecabtagene vicleucel)	B-cell maturation antigen (BCMA)-directed genetically modified autologous chimeric antigen receptor (CAR)-positive T cell immunotherapy	Relapsed or refractory large B-cell lymphoma	BCMA expressed by malignant plasma cells activates CAR-positive T cell proliferation, and subsequent plasma cell cytolysis	2021	Celgene Corporation, a Bristol-Myers Squibb Company	(22)
PROVENGE^®^ (sipuleucel-T)	Autologous cellular immunotherapy consisting of CD54^+^ cells activated with PAP-GM-CSF	Metastatic castration-resistant prostate cancer	CD54^+^ cells activated with PAP-GM-CSF take up, process, and express PAP, then induce an immune response against PAP-expressing prostate cancer cells	2010	Dendreon Corporation	(23)
GINTUIT (Allogeneic Cultured Keratinocytes and Fibroblasts in Bovine Collagen)	Allogeneic ready-to-use cellular scaffold consisting of human keratinocytes, fibroblasts, and extracellular matrix proteins, as well as bovine collagen	Surgically created vascular wound bed in the treatment of mucogingival conditions	Does not function as a tissue graft; increases keratinized tissue at the treated site in an unknown mechanism	2012	Organogenesis Inc.	(24)
LAVIV^®^ (azficel-T)	Autologous cellular product consisting of fibroblasts	Aesthetics of nasolabial fold wrinkles	Unknown	2011	Fibrocell Technologies, Inc.	(25)
MACI ^®^ (autologous cultured chondrocytes on porcine collagen membrane)	Cellular scaffold product consisting of autologous cultured chondrocytes on a resorbable porcine Type I/III collagen membrane	Symptomatic full-thickness cartilage defects of the knee	Unknown	2016	Vericel Corporation	(26)

## Stem Cell-Based Cell Therapies

### Overview of Stem Cells

Stem cells can be found in an organism in embryos and adult cells; they are a type of unspecialized, self-renewable cells prepped to differentiate into any cell type and/or as many cell types (28). What dictates how many cell types stem cells can differentiate into is their developmental potency. Developmental potency represents a differentiation continuum starting with totipotency (i.e., highest differentiation potential; e.g., zygote) and dwindling to pluripotency (e.g., embryonic stem cells), multipotency (e.g., hematopoietic stem cells), oligopotency (e.g., myeloid stem cells), and unipotency (i.e., least differentiation potential; e.g., dermatocytes) (29, 30). During passage along this continuum of potency toward mature/specialized cells, stem cells lose their self-renewal and differentiation potential (30). However, this hierarchy can be artificially reversed by nuclear reprogramming methods, including the use of transcriptional factors, which can eventually induce pluripotency in any cell type (31, 32). Stem cell specialization is influenced by external signals (e.g., physical contact between cells, paracrine secretions of nearby tissue, and tissue type), internal signals (e.g., genes), and epigenetics (embryonic cell origin) (29, 33). Depending on the type of stem cells, stem cell specialization can be detected by *in silico* gene expression analysis [e.g., PluriTest bioinformatic assay (34)] and validated by several techniques, including microarrays, polymerase chain reaction (PCR), and immunocytochemistry (35–38). Specific surface markers, molecular markers (e.g., transcription factors, microRNAs, transcription regulators, histone modifiers, DNA methylation state, X chromosome functional state, key molecular signaling pathways) (39–45), functional assays (e.g., teratoma formation assay, *in vitro* differentiation assay, blastocyst chimerism) (46–48), and culture characteristics (e.g., morphology, tolerance to single cell dissociation by trypsinization) (33) also help guide the evaluation of developmental potency.

### Stem Cells Used or Targeted by Cell Therapy

Stem cells used or targeted by cell therapy can be grouped into three categories: pluripotent stem cells (PSCs), adult stem cells (ASCs), and cancer stem cells (CSCs).

#### PSCs: Types and Use in Cell Therapy

PSCs give rise to all cell types except extraembryonic placental cells; they include embryonic stem cells (ESCs), found in the inner blastocyst cell mass of preimplantation embryos; epiblast stem cells (EpiSCs) and embryonic germ cells (EGCs), found in postimplantation embryos; and induced pluripotent stem cells (iPSCs), derived from direct reprogramming of postnatal/adult somatic cells *in vitro* (30, 33, 49). In 1981, Evans and Kaufman established the first murine PSC lines in culture following isolation of ESCs from mouse blastocysts *in vitro* (50). In 1998, Thomson and colleagues established the first human ESC line from *in vitro*-fertilized human embryos (51). In 2006, Yamanaka and Takahashi generated artificial PSCs (i.e., iPSCs) from adult and embryonic mouse somatic cells (fibroblasts) by induction with transcription factors (Oct-3/4, Sox2, KLF4, and c-Myc) (52). In 2007, Takahashi and colleagues used the same four transcription factors to generate iPSCs from adult human somatic cells (dermal fibroblasts) (32). Although ESCs and iPSCs have been proven to be molecularly and biologically equivalent, the use of ESCs is restricted due to ethical obligations related to endangering fetal lives (33). Generally, the clinical use of PSCs (elaborated further in section Clinical benefits of cell therapy by select fields) lacks therapeutic evidence and is limited to investigational regenerative medicine, with the rationale of cell differentiation/tissue repair in different diseases, including macular degeneration and heart failure (53–59).

#### ASCs: Types and Use in Cell Therapy

Somatic or ASCs are rare, undifferentiated cells distributed among differentiated or specialized cells in organs of a developed organism (60). With more limited self-renewal and differentiation potentials than PSCs, ASCs replenish lost cells or contribute to the healing or growth of cells by giving rise to precursor or progenitor cells and ultimately differentiated cells (61). ASCs include hematopoietic stem cells (HSCs), skin stem cells (SSCs), neural stem cells (NSCs), and mesenchymal stem cells (MSCs) (62). HSCs are mostly found in the bone marrow (BM) and give rise to all mature blood cells: red blood cells, white blood cells, and platelets (63). SSCs, such as epidermal stem cells and hair follicle stem cells, maintain skin integrity (64). NSCs are self-renewable stem cells found in the central nervous system and can give rise to nerve cells, oligodendrocytes, and astrocytes (65). MSCs are of mesodermal, non-hematopoietic origins and are present in multiple tissues, including BM, adipose tissue, peripheral blood, and placenta (66). They can differentiate into bone, cartilage, and fat cells, as well as cells of ectodermal or endodermal parentage (67, 68). Unlike ESCs, which are mainly defined by their origin in embryos using molecular and functional assays, ASCs have varying defining criteria, with cell morphology and surface markers being the go-to in most experimental evaluations (61). However, morphology and surface markers of ACSs are generally indistinguishable from those of mature cells; therefore, ASCs cannot be readily isolated from tissues, but enriched to varying degrees of purity in tissue extracts (60, 61). The use of ASCs (further elaborated in Section Clinical benefits of cell therapy by select fields) is mainly observed with HSCs or MSCs and envelops several clinical fields. For example, MSCs and HSCs are widely used in regenerative settings as, respectively, investigational and established modalities with the rationale of repopulating damaged cells or resetting tissue homeostasis (69–74). In immune system disorders, HSCs and MSCs have been generally used as investigational agents to alleviate disease activity with their vast mechanisms of action, and have shown varying success rates depending on the disease type (75–90). In cancer, HSCs have long been the standard treatment for hematological malignancies due to their regenerative potential (91); they have further been investigated in solid cancers as progenitors of immune cells, eventually driving tumor regression (92, 93). MSCs have also been investigated in cancer settings due to their anti-tumorigenic properties (66, 94, 95) yet have had only limited successes (96, 97).

Currently a hot topic in translational stem cell research, PSC- and ASC-derived organoids are highlight-worthy. Despite pending clinical investigations, these organoids hold promise as future regenerative medicine applications by offering *in vitro* three dimensional (3D) structural and functional mimicry of organs (98). Originally, these organoids are patient-derived stem cells manipulated and grown in controlled media formulations to dictate their differentiation, then propagated into 3D structures/matrices (99). Besides their potential in organogenesis and regeneration for cell-based therapy (100), PSC- and ASC-derived organoids represent useful tools for drug screening and disease modeling (98, 101). For instance, human PSCs have been used to grow lung organoids *in vitro*, with tissular and cellular compartmentalization similar to the native lung (102, 103). Similarly, kidney organoids structurally equivalent to the human fetal kidney were derived from human PSCs (104), further demonstrating native tissue-specific functions (105).

#### CSCs: A Therapeutic Target

CSCs, or tumor-initiating cells, are found within solid and blood tumors and originate from normal stem cells or progenitor cells by several proposed mechanisms, such as mutations, gene transfer, epigenetic alterations, and microenvironmental factors (106, 107). CSCs possess self-renewal, differentiation, metastasis, and immunosuppressive properties and play an important role in cancer growth, metastasis, relapse, and resistance to chemotherapy and radiotherapy (107, 108). Identification criteria of CSCs generally include surface protein markers (e.g., CD133, CD44, tumor-associated antigens) and metabolic/functional properties (e.g., high metabolism, slow cell division); however, they might overlap with those of normal somatic/germ cells or of other stem cells (49). Generally, the clinical use of CSCs (see Section Clinical benefits of cell therapy by select fields) is seen in cancer settings and involves targeting CSCs by different signaling pathway-interfering agents that subsequently prevent cancer growth and relapse (109, 110).

## Non–Stem Cell-Based Cell Therapies

Non–stem cell-based cell therapies are generally somatic cells that are isolated from the human body, propagated, expanded, selected, and subsequently administered to patients for curative, preventive, or diagnostic purposes (6). Non–stem cell-based cell therapies include fibroblasts, chondrocytes, keratinocytes, hepatocytes, pancreatic islet cells, and immune cells, such as T cells, dendritic cells (DCs), natural killer (NK) cells, and macrophages (5, 9, 111). Isolation techniques vary between somatic cells depending on their tissue localization and could include enzymatic digestion of harvested tissues (112) or processing of withdrawn blood (113). For example, peripheral blood mononuclear cells (PBMCs) can be collected by leukapheresis using automated systems, then cultured overnight, after which adherent cells (monocytes) can be separated from non-adherent cells (lymphocytes); DCs (HLA2DR^+^CD80^+^CD83^+^) can then be obtained by culturing adherent cells with granulocyte macrophage colony-stimulating factor, interleukin (IL)-4, and tumor necrosis factor over a week (114). Similarly variable are the characterization techniques of somatic cells, which are important to preserve a specific phenotype in sufficient yields, and can include microscopic examination, molecular analysis, immunocytochemistry, and gene expression analysis (112). Somatic cells are highly specialized (115) and can be further manipulated or treated before reintroduction into humans (116). Somatic cell-based therapies are generally employed as an *in vivo* source of enzymes, cytokines, and growth factors; as an adoptive cell therapy (ACT) to treat cancers; as transplanted cells, such as hepatocytes or pancreatic islet cells, to correct inborn metabolic errors; or as scaffold-based or -free cellular systems to treat ulcers, burns, or cartilage lesions (9, 117).

The application of cell grafts, such as hepatocytes, has only been slowly progressing due to technological hurdles and limited data supporting clinical efficacy and durability (111). For instance, hepatocyte transplantation has not yet been able to replace liver transplantation, due to limitations in post-transplant histological assessment and engraftment, despite limited clinical data showing its potential for being a future alternative to organ transplantation in treating patients with hepatic disease (118, 119). Contrarily, pancreatic islet cell transplantation for the treatment of insulin-deficient diabetes and pancreatitis has shown more promising potential (120), with clinical outcomes being dependent on islet availability and engraftment success rates, and limited with non-specific inflammatory/thrombotic mechanisms post-transplant. Indeed, in Canada, Australia, and several European countries, islet transplantation has become a standard of care for select patients (121).

ACT involves the intravenous transfer of modified peripheral or tumor-resident immune cells into patients to mount an immunologic reaction against tumors. Modified immune cells used in ACT include tumor-infiltrating lymphocytes (TILs), tumor-specific T-cell receptor (TCR)–modified T cells, and chimeric antigen receptor (CAR)-T cells (122). TILs can be grown from different tumor types under standard culture conditions *ex vivo*. Prior to TIL infusion, patients undergo lymphodepleting chemotherapy, and shortly after, they are administered high-dose IL-2 to amplify the therapeutic potency of TILs (123–125). T cells isolated from peripheral blood by leukapheresis can be genetically engineered *in vitro* to express modified TCRs that can be directed against specific tumor antigens, such as melanoma differentiation antigens and cancer/testis antigens (126); however, the downside of this TCR gene therapy remains its evasion by tumor cells, which can downregulate their major histocompatibility complex (MHC) expression (127). TCR gene therapy also generally involves patient preconditioning with lymphodepleting regimens and IL-2 support (128). CAR-T cells employ synthetic antibody-based CARs, which can be of a proteinaceous, carbohydrate, or glycolipid nature (124). The transfer of CARs to T cells can be performed by various techniques, including retroviral infection. The genetic construct of CARs encodes the single-chain variable fragment (scFv) of a monoclonal antibody (serves as the extracellular antigen recognitions domain), a CD3ζ chain (serves as the intracellular signaling domain of TCR), and a co-receptor, such as CD28, for co-stimulation (129, 130). Upon tumor antigen binding by the scFv domain, CD3ζ is phosphorylated, resulting in downstream signaling that is further amplified by co-receptor signals and that culminates in induction of cytotoxic activity (131). CAR-T cells are functionally similar to TCR gene therapy yet function in a non–MHC-restricted manner (132). Since their discovery in the 1980s (133), CAR-T cells continue to evolve. In 2003, second-generation CAR-T cells were redesigned to target CD19 in the setting of B-cell malignancies (134). Today, next-generation CAR-T cells explore innovative strategies aiming to improve antigen recognition, enhance cell proliferation and persistence, and evade the immunosuppressive tumor microenvironment (135, 136).

Other ACT strategies include lymphokine-activated killer (LAK) cells, cytokine-induced killer (CIK) cells, γδ T cells, and NK cells (137). LAK cells are PBMCs derived from patients by multiple leukaphereses and incubated with IL-2; they were first demonstrated in 1984 to possess antitumor properties (138). CIK cells are also a heterogenous mixture of lymphocytes (mostly CD3^+^CD8^+^CD56^+^ T cells) with natural killer T (NKT) cell phenotype generated by incubation with various types of molecules, such as IL-2, IFN-γ, and CD3 monoclonal antibodies; their antitumor capacity can be further elevated by incubation with other cytokines, including IFN-γ and IL-1β (139). γδ T cells constitute 5% of peripheral blood T-cell counts and are characterized by their expression of the γδ TCR instead of the more conventional αβ TCR (140); following their *ex vivo* expansion, γδ T cells become tumor-reactive with strong, non–MHC-restricted cytotoxicity (141). Like γδ T cells, NK cells possess the ability to kill tumor cells in a non–MHC-restricted manner. Immunophenotypically, NK cells are CD3^−^CD56^+^ (137, 142). To enhance their antitumor activity, NK cells are expanded by IL-2 incubation and/or co-administration (143, 144).

## Multicellular Therapies

The term “multicellular therapies” is coined by few sources and can be defined as therapies containing at least two stem cell and/or non-stem cell types cultured from isolated cells or tissue extracts (145–147). The generation of multicellular therapies involves selective phenotypic expansion, rather than purification or enrichment processes, and can exploit automated cell-processing technologies (145, 147, 148). The distinct cell constituents of a multicellular therapy possess a broad range of biological activities, which contribute to its typically abstruse mechanism of action (26, 145). Therefore, the composition and/or functional intricacies of multicellular therapies might mirror those of normal tissues (148). Examples of these therapies include ACT products (149), scaffold-based or -free cellular products (9), stromal vascular fraction (SVF), stem cell transplant (150), and bone marrow aspirate (BMA)-derived therapies (151).

### ACT Products

Among ACT products, TILs is a multicellular therapy that includes different lymphocyte lineages, including T cells and B cells (149). In cancer biology, lymphocytes recognize growing cancer cells and infiltrate the tumor. Once in the tumor, TILs try to initiate cancer killing. However, cancer cells can inactivate TILs to evade immunosurveillance by ligating their checkpoint receptors [e.g., programmed death 1 (PD-1), cytotoxic T lymphocyte-associated protein 4 (CTLA-4)], which are normally bound by specific ligands (e.g., programmed death-ligand 1 (PD-L1), B7) to control immune activity (152). Many tumors express PD-1 receptor ligands, such as PD-L1, permitting the mitigation of anti-tumor immunity (153, 154). Therefrom, immune checkpoint inhibitors were devised to lift off immune cell suppression and promote anti-cancer immunity (155). Indeed, it has been shown that TILs can promote tumor invasion and metastasis through several mechanisms, including cancer cell-leukocyte fusion and recruitment of regulatory T cells (Tregs) (156). The 1970's recorded the first attempts of lymphocyte isolation from tumor tissues (157, 158). In the next decade, IL-2-expanded isolated TILs showed significant antitumor activity *in vivo* (159), as opposed to TILs alone (160). TIL preparation involves tumor excision, digestion, culture with IL-2, and assessment for specific tumor recognition; tumor-specific TIL cultures are then expanded using anti-CD3 monoclonal antibody, high IL-2 concentrations, and irradiated allogeneic feeder cells (161). Characterization methods of TIL cellularity include gene expression analyses and analytical tools, such as CIBERSORT (162, 163). TIL products are heterogenous in terms of CD8^+^/CD4^+^ T cell ratios and T-cell differentiation stage and can be impacted by tumor biology (164). For an endowment of resistance to tumor suppression and/or enhanced tumor homing, TILs can be genetically modified either using different types of vectors or *via* gene editing technologies (165).

LAK cells are another ACT product composed of IL-2-activated PBMC's, mainly NK cells, NKT cells, and T cells, with non-specific cytotoxicity and non–MHC-restrictive cytotoxic effects (137). The use of LAK cells is limited to few cancer types due to their difficult amplification and associated adverse effects (166, 167). LAK cells have been reported to induce tumor cell killing by releasing cytolytic mediators, including perforin and granzymes (168).

CIK cells are a subset of T lymphocytes with an NKT cell phenotype, and can be expanded *ex vivo* from PBMC's or BM mononuclear cells. When activated, CIK cells stimulate the immune system to recognize and eradicate tumor cells in a non–MHC-restricted manner (137, 139).

### Scaffold-Based or -Free Cellular Products

Scaffold-based cellular products are engineered technologies that deliver different cell types (e.g., fibroblasts and keratinocytes) seeded within 3D biocompatible tissue analogs (9). Traditionally, scaffold-based cellular products employ biodegradable natural or synthetic polymers (e.g., bovine collagen, hydrogels, sponges) with sophisticated porous networks through which oxygen, nutrients, and metabolites can be exchanged (169, 170). Current scaffold-based cellular products with FDA approval are used for the treatment of diabetic foot ulcers (e.g., Apligraf^®^, Dermagraft^®^), burns (OrCel^®^), and mucogingival conditions (GINTUIT) (9, 24, 171, 172). Scaffold-free cellular products are tissue analogs that are densely populated with cells carried and protected by their secreted, tissue-specific extracellular matrix (ECM) (9). This biotechnology can employ temperature-responsive polymers (e.g., pNIPAM, PVME) that transition between hydrophobic and hydrophilic states at certain temperatures, allowing the control of cell culture and growth and subsequently the deposition of ECM and the formation of cell sheets that adhere to biological surfaces (173–175). Several automated technologies (e.g., robots, bioreactors) can also be used to enhance the scalability, elevate the architectural biomimicry, or allow for the perfusion of these tissue analogs (176–178). An example of commercially available, FDA-approved scaffold-free cellular products is Epicel^®^ (cultured epidermal autografts), a petrolatum gauze composed of sheets of autologous keratinocytes and proliferation-arrested murine fibroblasts and indicated for deep/full burns (179). Generally, the specific mechanisms of action of scaffold-based or -free cellular products are unknown, but are surmised to involve the production of cytokines and growth factors similar to healthy human skin (180). Although these products represent important advances in regenerative medicine, they are still limited by their high costs and non-regenerative outcomes, including their inability to fully reconstitute the damaged skin architecture (181).

### SVF

SVF is a heterogeneous mixture of stromal and vascular cells, including ASCs, granulocytes, monocytes, lymphocytes, pericytes, and endothelial progenitor cells (EPCs), obtained from the processing of adipose tissue (e.g., lipoaspirate, excised fat) (182–185). Besides its use as an investigational product in different clinical settings (186–188), SVF is used as a source to isolate ASCs (i.e., adipose-derived stem cells, or ADSCs), which can constitute up to 10% of its fraction depending on the processing technique, usually involving serial straining and centrifugation of SVF and cell culture in growth media (189). SVF composition can be identified immunophenotypically by flowcytometry, for example for the presence of ADSCs (CD45^−^CD235a^−^CD31^−^CD34^+^), which share several surface markers with BM-MSCs but are CD36^+^CD106^−^. Other techniques used for identifying the cellular composition of SVF include lineage-specific differentiation assays and biochemical or PCR evaluation (190). Due to its heterogeneous composition, SVF functions in various mechanisms, including paracrine signaling through cytokines, chemokines, and growth factors and cell-cell interactions, ultimately promoting neovascularization, cell repair, and immunomodulation (191).

### Stem Cell Transplant

Stem cell transplant is performed in settings that damage the body's stem cells, including hematologic malignancies (e.g., leukemia, lymphoma, multiple myeloma, neuroblastoma) or cancer therapy (e.g., high-dose chemotherapy, total body irradiation) (192). Stem cell transplant relies on 3 stem cell sources: bone marrow, peripheral blood, and umbilical cord blood (193).

Stem cell transplant with bone marrow as the source of stem cells is known as bone marrow transplantation (BMT), which has been in practice since the 1960's (194). BMT entails BM aspiration (195) for harvesting HSCs (196) as well as progenitor cells, MSCs, lymphocytes, neutrophils, platelets, red blood cells, eosinophils, basophils, and monocytes (197, 198) (see Section BMA-derived therapies). Peripheral blood stem cell transplantation (PBSCT) is another type of hematopoietic stem cell transplantation (HSCT) that uses peripheral blood-derived HSCs (199). PBSCT came forth in the 1990's (196) as an alternative to bone marrow transplantation (BMT) (194), due to easier stem cell collection, higher stem cell yields, and faster patient recovery post-transplantation (150). In this procedure, autologous or HLA-matched allogeneic peripheral blood stem cells (PBSCs) are infused into the patient's bloodstream following a preparative conditioning regimen consisting of chemotherapy with/without total body irradiation that ensures immune tolerance of the engraftment. Once in the blood, PBSCs home toward the BM to repopulate lost blood cells or allow cancer remission (150, 200). PBSCs are collected by continuous-flow apheresis after mobilization using medications including granulocyte colony-stimulating factor (G-CSF) agents and chemokine receptor 4 (CXCR4) blockers (e.g., plerixafor); chemotherapy can also be used for mobilization (i.e., chemoembolization) (200). PBSCs are generally identified and quantified using flowcytometry *via* their immunophenotypic patterns (e.g., CD34^+^CD38^−^) (192). Besides CD34^+^ HSC subpopulations, PBSC grafts contain nucleated cells including DCs, T cells, B cells, NK cells, and monocytes (201). Compared to BM-derived stem cells, PBSCs express more lineage-specific differentiation antigens, are less metabolically active, and show higher clonogenicity (202). However, the clinical benefit/risk ratio of PBSCs vs. BM-derived stem cells is disputed, with the preference being dependent on the type of hematologic disease, the age of donors/recipients, and whether the HLA-matched donor is related or unrelated—factors which influence the incidence of graft vs. host disease (GVHD) or the patient's quality of life (196, 199). The differences in cellular composition (e.g., CD34^+^ and lymphocyte numbers) between both stem cell grafts is also associated with differences in their clinical outcomes (203).

Another source of stem cell transplantation is cord blood (CB), whose HSCs and hematopoietic progenitor cells are observed to differ from those of peripheral blood and BM in terms of surface markers, recovery speed of blood cells post-transplant, clinical outcomes, and GVHD incidence (204, 205). As of 2011 to date, eight allogeneic cord blood products have gained FDA approval for the treatment of hematopoietic system disorders. These products mainly contain HSCs and hematopoietic progenitor cells, which migrate to the BM where they divide, and their progeny cells mature and subsequently replace lost blood cell reservoirs. Notable, these products are also composed of monocytes, lymphocytes, and granulocytes, which render their mechanisms of action only partially known (10–17).

### BMA-Derived Therapies

BMA-derived therapies are commonly termed concentrated bone marrow aspirate (cBMA), bone marrow concentrate (BMC), or bone marrow aspirate concentrate (BMAC) (151). BM aspiration is a procedure performed under local or general anesthesia, in which a liquid sample is collected from the BM of usually the anterior or posterior iliac crest among other bones (195). Since the early 1960's, the BM has been the chief source for harvesting HSCs for BMT procedures (196); however, its use in this context has diminished following the emergence of PBSCT (206, 207). BMA includes various cell types including HSCs, progenitor cells, MSCs, lymphocytes, neutrophils, platelets, red blood cells, eosinophils, basophils, and monocytes (197, 198). The processing of BMA to concentrate nucleated cell yields, such as MSCs which represent 0.001% of the non-hematopoietic, multipotent cellular portion of BMA, yields BMAC (198). The concentration of BMA can be performed by different techniques, including automated centrifugation systems (208) or cell filtration systems (209). In BMAC, concentrations of nucleated cells become 5-fold higher, and concentrations of MSCs 6-fold higher (210, 211). The composition of BMAC also includes HSCs, progenitor cells, white blood cells, platelets, and cytokines/growth factors (212, 213) and can be characterized by microscopy, flowcytometry, cytogenetic and molecular analyses, and cytochemical staining (214). Generally, the clinical application of BMAC spans orthopedic settings, in which it can be sterilely injected intra-articularly under the guidance of fluoroscopy or ultrasonography (215). Like SVF, BMAC functions in various mechanisms involving paracrine signaling by MSCs and nucleated cells that drive tissue repair and immunomodulation (66, 209, 210) and by growth factors and cytokines that induce tissue growth and promote reparative processes (198, 216).

## Acellular Therapies With Multicellular Components

Platelet-rich plasma (PRP) is an anticoagulated blood product obtained by differential centrifugation of whole blood and predominantly contains platelets in concentrations that can exceed up to 5 times physiologic platelet concentrations (217, 218). Platelets are acellular fragments derived from maturing megakaryocytes and function mainly in maintaining primary hemostasis and thrombosis to preserve vascular integrity (219). Although predominated by platelets—reservoirs of multitudinous immunologic molecules, soluble proteins, and growth factors (220)— and plasma components, PRP contains cellular components, such as leukocytes (217). Several commercially available kits can be used for PRP processing with varying outcomes in terms of platelet, red blood cell, and leukocyte concentrations (221). PRP composition can be subsequently analyzed using various analytical methods, such as automated hematology analyzers, microscopy, flowcytometry, and spectrophotometry (222, 223). Because of their unstandardized preparation protocols yielding heterogeneous formulations, PRP products can be further classified in clinical settings on the bases of platelet concentrations/activation and cellularity using various non-consensual classification systems, including the PAW, the PLRA, and the International Society on Thrombosis and Hemostasis systems (221, 224). PRP functions in several mechanisms driven by cytokines, growth factors, platelets, and nucleated cells, altogether which exert anti-inflammatory effects and promote tissue repair (225, 226).

## Regulatory Considerations for Cell Therapy

For manufacturers, as well as researchers and clinicians, it is important to be aware of the FDA's regulatory guidance on cell therapy products. Human cells, tissues, and cellular and tissue-based products (HCT/P) are defined by the FDA under the Title 21 of the Code of Federal Regulations (CFR) Part 1271.3(d), or [21 CFR Part 1271.3(d)], as “articles containing or consisting of human cells or tissues that are intended for implantation, transplantation, infusion, or transfer into a human recipient.” Falling under this definition are several examples, including HSCs/progenitor cells derived from peripheral blood or CB, manipulated autologous chondrocytes, and epithelial cells on a synthetic matrix. If the therapy does not meet the definition of HCT/P in 21 CFR 1271.3(d), such as blood components/derivatives (e.g., PRP) and minimally manipulated BMA, the regulations in 21 CFR Part 1271 do not apply (227).

For therapies meeting the definition of HCT/P in 21 CFR 1271.3(d), the 21 CFR 1271.15(b) further guides how HCT/P are regulated. The “same surgical procedure (SSP) exception” in 21 CFR 1271.15(b) states that it is not required to comply with the requirements in 21 CFR Part 1271 if the establishment is collecting and administering the HCT/P autologously, within the same surgical procedure, and in their original form (if processed, only rinsing, cleansing, sizing, and shaping are allowed) (227, 228). Otherwise, the algorithm progresses to the requirements of 21 CFR 1271.10(a). The criteria under this title specify that the HCT/P is minimally manipulated; intended for homologous use; not combined with other active agents; without a systemic effect; and—if with a systemic effect—administered autologously or to first-/second-degree blood relatives (227). If these criteria are met, the FDA allows the use of the cell therapy in the framework of regulatory guidelines governing disease transmission, yet without premarket approval/biologics license application, solely under section 361 of the Public Health Service (PHS) Act and regulations in 21 CFR Part 1271. Otherwise, if the cell therapy does not meet the criteria under 21 CFR 1271.10(a), it is regulated as a biological product under the Federal Food, Drug, and Cosmetic (FD&C) Act and/or section 351 of the PHS Act and applicable regulations. In this case, the cell therapy would require premarket approval, and the establishment needs to register the therapy and apply for a biologics license for lawful marketing, or have an investigational new drug (IND) application in effect if the therapy is investigational (227, 228).

The importance of being well informed about the above regulations becomes more obvious with the recent aggressive enforcement the FDA has begun to undertake to protect patients from risks of unapproved products being otherwise dispensed as HCT/P falling under section 361 of the PHS Act and regulations in 21 CFR Part 1271. In May 2018, the FDA initiated an action against a stem cell clinic for administering non-compliant autologous SVF to patients. On June 3, 2019, the Florida court ruled in favor of the FDA because, according to the FDA arguments, the isolated SVF no longer represented adipose tissue (i.e., not in its original form, or adipose tissue) after removal from the patient, and because the therapeutic use of the SVF differed from the natural function of adipose tissue (i.e., not intended for homologous use). Although the stem cell clinic argued that CFR 1271.15(b) and 21 CFR 1271.10(a) apply to their SVF product, the court saw otherwise, considering the case as a violation of federal laws and the product as a “drug” falling under the FD&C Act and necessitating extensive pre-approval. On June 25, 2019, the court ordered the stem cell clinic to cease its offering SVF services until further FDA compliance. In addition to that SVF clinic, the FDA has issued multiple warnings to other clinics working with stem cells and umbilical cord-derived products (229, 230). Counterintuitively, a California federal judge has denied a government motion initiated simultaneously with the former lawsuit, against a stem cell treatment center. In the trial, the FDA argued that the center was using illicit SVF therapies that are manipulated prior to implantation to treat degenerative diseases. The manipulation according to the FDA occurred in the removal step of the adipose tissue, thus generating an SVF product to which the SSP exception does not apply. The defendant countered that their SVF is unaltered, despite the removal of adipose tissue, and thus complies with all requirements in 21 CFR Part 1271 (231). In the hearing (232), the judge considered that the SSP exception is unambiguous and read that it does not require the reimplantation of all the removed tissue, much like coronary artery bypass procedures in which surgeons do not implant the removed blood and excess artery. Based on this logic, the judge considered that the SSP exception applies to the SVF product, whose natural host tissue was removed as part of the collection process without further alteration to the SVF content. The court saw that the FDA's interpretation of the SSP exception is “unreasonable and creates enforcement inconsistency” and considered that “the agency's reading must fall within the bounds of reasonable interpretation… a requirement an agency can fail.” (232) These incidents are indicative that the FDA regulations governing the use of cell therapy products are confusing and might not be safe from misinterpretations or dispute.

On another note, other multicellular products, such as BMAC, have not received FDA warnings and continue to be used as HCT/P under section 361 of the PHS Act and regulations in 21 CFR Part 1271.

## Clinical Benefits of Cell Therapy by Select Fields

### Regenerative Medicine

Regenerative medicine deploys a body's own cells and growth factors to repair tissues by restoring their lost functions (111). Several cell therapies in regenerative medicine have become either established practices or commercially available with FDA approval, such as keratinocyte- and/or fibroblast-derived skin substitutes for treatment of diabetic foot ulcers (172, 233) or burns (179); keratinocyte- and fibroblast-containing scaffold products for treatment of surgically created vascular wound beds in the oral cavity (24); fibroblast intradermal injections for improvement of appearance of nasolabial fold wrinkles (25); chondrocyte-containing scaffold implants for treatment of knee cartilage defects (26); and cord blood-derived HSC/hematopoietic progenitor cell products for treatment of hematopoietic system disorders that are inherited, acquired, or result from myeloablative treatment (10–17). Although commercial cell therapies are beneficial in repairing tissues, they are unable yet to regenerate them (234). Clinical development is also an arduous process that hinders the introduction of new products into the market (235, 236). This can be seen in the proportion of approved biologics over a 9 year period, which reached 23% of all approved drugs. Additionally, biologics in the US are granted 12 years of exclusivity protection vs. ~7 years for new chemical entities (237).

In clinical investigation settings, multiple cell therapies, as well as acellular therapies with cellular components, have been assessed for their safety and efficacy in a regenerative context, including PRP, ESCs, iPSCs, SVF, ADSCs, MSCs, and BMAC (190, 234, 238, 239).

PRP is widely evaluated in orthopedics due to its enriched composition of cytokines, growth factors, and platelets, which establish an anti-inflammatory environment at the site of injection and promote skeletal and connective tissue regeneration and reconstruction (225, 226). For instance, PRP preparations have demonstrated efficacy and safety in tendon injuries (240, 241), rotator cuff tears (242), osteoarthritis (OA) of the knee or hip (243, 244), and muscle injuries (245), with benefits being mostly symptomatic relief. The cellularity of PRP can also dictate clinical outcomes, thus classifying PRP preparations into leukocyte-rich PRP (LR-PRP)—with leukocyte concentrations exceeding baseline levels—and leukocyte-poor PRP (LP-PRP) —with leukocyte concentrations below baseline levels (241). Accordingly, it is recommended that PRP be analyzed for its leukocyte content and used in accordance with the catabolic vs. anabolic requirements of the treated condition (246).

The clinical application of ESCs is restricted by ethical concerns, regulatory bodies, and the lack of preclinical evidence supporting their use (53, 54). However, few successful outcomes in regenerative medicine merit acknowledgment. For example, human embryonic stem cells (hESCs) have improved the vision of patients with macular degeneration and macular dystrophy by differentiating into photoreceptors and retinal pigment epithelial cells (55). In a case report, cardiomyocytes derived from hESCs have also improved the ejection fraction of a 68 year old patient with severe heart failure and without inducing subsequent complications (56).

Despite presenting several advantages over ESCs (e.g., non-invasive collection, less immune rejection, ethically unrestricted nature), the use of iPSCs in clinical settings is still farfetched due to lack of therapeutic evidence as well as other preparation and standardization obstacles (57). Indeed, a data compilation in 2018 showed that the fraction of clinical trials investigating the aptness of iPSCs as a treatment modality constitutes only 11% of the total clinical trials of iPSCs, including those terminated (58). The first and potentially only reported clinical benefits of iPSC-based therapy are minimal and date to 2017 in a patient with neovascular age-related macular degeneration (59). On the other hand, iPSCs—like ESCs—have been extensively employed as research tools for drug toxicity testing (e.g., drug-induced QT prolongation) and—unlike ESCs—have been useful for disease modeling and drug discovery studies (247).

SVF was first clinically investigated in reconstructive surgeries with the rationale of promoting adipose tissue survival and thus provide structural tissue support (248). Multiple other studies followed, in which SVF was investigated for its healing and regenerative abilities. For example, SVF has promoted neovascularization and improved tissue hydration in patients with radiotherapy-induced lesions, owing partially to its ADSC composition (249). Few other examples of regenerative settings in which SVF has provided patients with clinical benefits include knee OA (250, 251), chronic wound healing (252), urogenital conditions (253), and systemic sclerosis (SS)-associated facial handicap (254).

In regenerative medicine, ASCs are considered the most promising among cell therapies, and ADSCs constitute an ideal option due to their ease of harvest requiring minimal invasiveness; multi-lineage differentiation potential; and anti-inflammatory and proangiogenic secretome (255). To date, there are 11 active or recruiting registered studies involving ADSCs as an intervention in conditions such as knee OA and chronic kidney disease (256). Data disclosed hitherto, mostly by pilot studies, show that—despite not living up fully to their regenerative rationale—ADSCs have shown a promising potential in multiple settings, including ischemic heart disease (73, 74), acute myocardial infarction (257), knee OA (258, 259), peripheral vascular disease (260), SS-associated ulcers (261, 262), ischemic diabetic feet (252), urogenital conditions (263, 264), and breast cancer-associated lymphedema (265). For example, an early-phase placebo-controlled trial has shown that the intracoronary infusion of ADSCs is well tolerated, improves left ventricular ejection fraction (LVEF), and reduces infarct size after 6 months of follow-up in patients with acute myocardial infarction (257). Similarly, the intramyocardial delivery of ADSCs has been shown to significantly improve LVEF and exercise tolerance in patients with chronic ischemic heart disease (73, 74).

Among ASCs, MSCs are gaining considerable attention as a cell therapy intervention in human studies, especially with the production of good manufacturing practice (GMP)-compliant human MSCs (66, 266). To date, there are at least 180 active or recruiting registered studies involving MSCs as an intervention in various conditions (69). In regenerative settings, MSCs have exhibited a promising potential in osteogenesis imperfecta (70), Crohn's disease (71), deep burns (72), periodontal defects (267), chondral/bone defects (268, 269), and diabetic foot (270). The benefits of MSCs in clinical investigations are mostly linked, not to their multi-lineage differentiation potential, but rather to their secretome, which establishes a nutritive microenvironment, promoting autocrine and paracrine signaling that inhibits apoptosis and dictates angiogenesis, local tissue mitosis, and cross-communication with resident stem cells (66, 271–273).

BMAC has emerged as a potential alternative for regenerative therapy, owing mainly to its enriched composition of growth factors and MSCs among other cell types (216). In regenerative settings—mainly orthopedics—BMAC has demonstrated a promising potential, as it provided patients with clinical benefits and/or improved diagnostic imaging outcomes of patients (209, 274, 275). To date, there are at least 14 active or recruiting registered studies involving BMAC as an intervention mainly in orthopedic conditions (276). The mechanism of action of BMAC remains unclear, and no serious attempts have been made to delineate the interactions between the different components of BMAC, which might collectively be at the origins of BMAC outcomes (277, 278). Among the components theorized to contribute to the therapeutic potential of BMAC are MSCs, which are endowed with tissue function-enhancing regenerative and immunologic properties (66, 209, 210); growth factors, which promote tissue growth; and nucleated cells (e.g., lymphocytes), which secrete various reparative cytokines and growth factors that act via paracrine pathways (198, 216). However, the conclusions drawn by these reports about the role of BMAC components—specifically MSCs—in driving clinical outcomes are based on rather extrapolations than benchwork. Hence, further molecular investigations are necessary to fully understand the degree of contribution of each of these components in the observed orthopedic benefits. Noteworthy, BMAC could still become an established therapy even with a partially understood mechanism of action and without having to erroneously suggest that its beneficial outcomes are driven by MSCs. This possibility could be observed with HSCT, which has become an established therapy for treating immune diseases despite its elusive mechanism of action (75, 89).

### Immune System Disorders

Most immune system disorders develop due to excessive immune responses or autoimmune attacks (279). Primary treatment thus aims to alleviate inflammation, minimize symptoms, and prevent relapse (280). The rationale of exploiting cell therapy in immune system disorders extends beyond immune suppression and symptomatic relief to immune system resetting as a permanent cure (281). As of the late 1990's, BMT/HSCT has become the most established cell-based therapy for treating immune system disorders (75). HSCT has been shown to elicit durable outcomes in severe SS with acceptable rates of transplant-related mortality (76). In controlled phase 2/3 trials, HSCT has demonstrated efficacy in patients with autoimmune disorders, resulting for instance in 79% improvement in the disability status and marked improvement in disease relapse rates, MRI lesions, and quality of life in patients with multiple sclerosis (83). HSCT, including PBSCT, has also alleviated disease activity and stabilized/reversed organ dysfunction in patients with systemic lupus erythematosus (SLE) (84–86). While the benefits of HSCT in SLE are mostly reported by retrospective studies, prospective trials are limited and have not found significant benefits (83). On the other hand, HSCT has only shown transient responses or partial benefits in other immune diseases, such as rheumatoid arthritis (RA), vasculitis, and Crohn's disease (87, 88). Despite its benefits seen in most immune system disorders, HSCT's underlying mechanism of action remains elusive, with non-specific benefits being also omnipresent and pertaining to the accompanying regimen of lymphotoxic chemotherapy that reduces autoreactive antibodies (89). MSCs have also had their share of clinical successes in immune system disorders, specifically in GVHD, amyotrophic lateral sclerosis, and Crohn's anal fistula (77–81, 90). These benefits are linked to the immunomodulatory actions of MSCs originating mostly from their immune inhibitory secretome (82). Although several MSC-based therapies are approved worldwide for the treatment of immune diseases (including in Canada and Japan), they have not yet received FDA approval (66). Albeit to a less documented extent than BM-MSCs, ADSCs have also shown a promising potential as a cell therapy for the treatment of immune system disorders, such as GVHD, Crohn's disease, psoriasis, and SS (282–285). Other cell-based therapies with less reported benefits in immune diseases include PRP, which has been shown to reduce pain and inflammation with ultrasound imaging evidence in patients with RA (286), and Tregs, which have been shown to reduce the incidence of acute GVHD (287). DCs are another type of immunotherapies exploited in the treatment of patients with immune system disorders. For instance, tolerogenic DCs—a type of immature DCs that induce T-cell anergy and Treg differentiation causing peripheral tolerance (288)—have been reported to stabilize disease (289) or reduce inflammation and disease scores in RA (290). Other somatic cell-based therapies like pancreatic islet cell transplantation have resulted in substantial benefits in type 1 diabetes. For example, a single-arm phase 3 trial has shown that pancreatic islet transplantation leads to glycemic control and protection against severe hypoglycemic events in patients with type 1 diabetes (120). Therefore, it has become recommended globally that pancreatic islet transplantation be considered for patients whose problematic hypoglycemia persists despite insulin infusion or glycemic monitoring (291). Notable, clinical outcomes with pancreatic islet cell transplantation have been found to be associated with islet availability and engraftment success rates, which are elevated for instance in allogeneic transplants, where islets can be isolated from multiple donors (121). Other immunotherapies like CAR-T cells have not yet been reported to provide clinical benefits in immune conditions (292), despite promising preclinical outcomes (293).

### Cancer

The rationale of cancer therapy has evolved from the systemic targeting of tumors with chemotherapy/radiotherapy to a more targeted approach using novel biologic treatments, including monoclonal antibodies, oncolytic viruses, and cell therapy, such as antigen presenting cell (APC)-based anticancer vaccines and CAR-T cells (294). Another therapeutic approach in patients with cancer is the aforementioned regeneration of immune effectors specifically in hematological malignancies, in which case HSCT has long been the standard treatment (91). The year 2010 witnessed the FDA approval of PROVENGE^®^ (sipuleucel-T), the first and only APC-based anticancer vaccine indicated for the treatment of metastatic castration-resistant prostate cancer (23). Later in 2017, KYMRIAH™ (tisagenlecleucel) became the first CAR-T cell therapy to receive FDA approval for the treatment of relapsed or refractory B-cell precursor acute lymphoblastic leukemia and large B-cell lymphoma (19). Following tisagenlecleucel's steps, other autologous, CD19-directed CAR-T cell therapies then entered the market with the indication of treating relapsed or refractory large B-cell lymphoma or mantle cell lymphoma (Table 1) (18, 20, 21). In 2021, ABECMA^®^ (idecabtagene vicleucel) became the first B-cell maturation antigen (BCMA)-directed CAR-T cell therapy to receive FDA approval for the treatment of relapsed or refractory large B-cell lymphoma (22), with distinctive selectivity conferred by the highly selective expression of BCMA by malignant plasma cells (295).

Besides commercial cell therapy products, a multitude of cell therapies have been investigated for treating cancer in clinical settings. Among APC-based anticancer vaccines, DC-based anticancer vaccines—either created with primary CD1c^+^ myeloid DCs or engineered by fusion with patient-derived tumor cells, pulsation with tumor peptides/lysate, or electroporation with tumor associated antigen-encoding mRNA—have elucidated promising immunologic and/or clinical responses in B-cell lymphoma (296), multiple myeloma (297), acute myeloid leukemia (298), glioblastoma (299), and metastatic melanoma (300–302). CD34^+^ HSC-derived modified/manipulated DCs have also been clinically investigated in cancer settings with promising outcomes, such as generation of tumor-specific immunity and/or induction of tumor regression in patients with metastatic melanoma (92, 93). Using chemicals like polyethylene glycol, autologous primary DCs can be fused with irradiated, resected tumor cells to create tumor-DC hybrids whose subsequent bioengineering and administration to patients with glioblastoma receiving standard chemotherapy has been shown to improve clinical responses (303). Similarly, the vaccination of patients with acute myeloid leukemia who achieved remission following chemotherapy using autologous primary DCs fused with autologous cancer cells has led to the expansion of tumor-reactive T cell subsets and prolonged remission (304).

Investigational CAR-T cells have also shown high antitumor activity in relapsed/refractory multiple myeloma by targeting BCMA (305). Unlike CD-19- or BMAC-directed CAR-T cell therapy for hematologic malignancies, CAR-T cells directed against solid tumor antigens, such as PD-L1 and prostate-specific membrane antigen (PSMA), have had less clinical success due to obstacles pertaining to the suppressive nature of the tumor microenvironment and therapy persistence within the tumor (306). In small-scale studies, the use of bispecific CAR-T cells directed against CD19/BCMA in multiple myeloma has been met with promising patient responses (307). Among ACT, TCR-modified T cells directed against tumor-specific antigens have also had promising outcomes in cancer therapy, as they induced cancer regression in patients with melanoma (308, 309) and reduced metastases in patients with synovial cell sarcoma (310, 311). Similarly, TILs and LAK cells have been reported to induce tumor regression in patients with metastatic cancers (312–314). Additionally, LAK cells have improved the survival of patients with melanoma and patients with glioblastoma (315, 316), and TILs have augmented the rates of objective clinical responses of patients with metastatic melanoma (317). CIK cells were also reported to reduce disease recurrence or improve overall survival in patients with hepatocellular carcinoma and to augment the progression-free survival and overall survival in patients with renal cell carcinoma (318–320). The mechanism of action of CIK cells is observed to involve perforin-mediated tumor killing (321). On the other hand, although few phase 1 clinical trials have demonstrated the benefits of γδ T cells as a cancer immunotherapy, other studies have reported contrasting outcomes, revealing the suppressive façade of this T-cell subset and linking its presence within the tumor microenvironment to negative outcomes (137). What's more in ACT, allogeneic NK cells have only provided modest benefits to patients with acute myeloid leukemia (322) and patients with recurrent ovarian and breast cancer (323), generally due to their inhibition by host Tregs and/or the tumor as well as the high toxicity of IL-2 (137). In combined cell therapy approaches, CIK cells and tumor lysate-pulsed DCs infused intravenously at different time intervals have been shown to significantly prolong the median survival time at a rate comparable to chemotherapy in patients with colorectal cancer (114) and improve the overall survival and the quality of life in patients with advanced colorectal cancer (324). Similarly, DC-CIK immunotherapy has been reported to significantly prolong the overall survival and improve the quality of life in patients with advanced non-small cell lung cancer (325). Other combinatorial approaches include tumor lysate-loaded DC and TIL immunotherapy, which has revealed a promising potential based on evaluating objective clinical responses in a phase 1 study in patients with advanced melanoma (326). Patients with metastatic melanoma have also experienced immunologic responses and tumor regression upon treatment with the combined therapy comprising TCR-modified T cells directed against melanoma-associated antigen recognized by T cells (MART-1) and DCs (327).

Albeit to a less documented extent compared to ACT products or other cellular therapies, genetically engineered MSCs have been investigated as a cancer treatment due to their ease of obtainment and demonstrated tumor tropism and anti-tumorigenic properties; however, no benefits have been reported to date with patient-administered MSCs (66, 94), possibly due to their insufficient cell homing to tumors as reported in a phase 1 study (95) or their paradoxical pro-tumorigenic potential seen in several preclinical studies (66, 328). Noteworthy, based on previous promising preliminary data (329, 330), the first-in-human, first-in-child clinical trial for Celyvir—an autologous MSC-based therapy carrying oncolytic adenoviruses—has reported disease stabilization in two pediatric patients with neuroblastoma (96). Following the steps of this trial, other groups are exploring the potential of bioengineered MSCs carrying oncolytic viruses—viruses that evade immune surveillance and can conditionally replicate in tumor cells, unlike traditional attenuated viruses (97)—in patients with glioblastoma (NCT03896568) and patients with ovarian carcinomas (NCT02068794). Finally, several CSC-targeting agents for cancer treatment have been approved (e.g., vismodegib, ivosidenib, venetoclax) or are still under investigation, with mechanisms of action entailing the involvement with CSC pathways (109, 110).

## Multicellular vs. Unicellular Therapies

Multicellularity in an organism enables cell-cell communication, which is crucial during the different phases of tissue development starting early in embryogenesis and persisting through later regenerative processes (331). In regenerative medicine, there is a growing notion that a mixture of cell types rather than one cell type is important to promote long-term tissue repair driven by complex, poorly understood multicellular interactions typical of the physiological nature of organisms (277, 278). Compared to ADSCs, a mixture of ADSCs, EPCs, and lymphocytes among other cell types (i.e., SVF) has received more praise in preclinical comparative studies, in part due to the heterogeneity of cellular composition perceived to exploit more physiologic properties (e.g., angiogenesis, immunomodulation, cellular differentiation) that collectively drive better outcomes (332–335). Notable, the first study to compare MSCs vs. BMAC (i.e., MSCs, progenitor cells, white blood cells, etc…) on the scale of clinical and magnetic resonance imaging (MRI) outcomes in 52 patients with chondral knee defects has found no differences in retrospect between both treatments over a 2 year follow-up (336). Similarly, a recent retrospective study found no differences in postoperative radiological findings and pain/functionality outcomes in patients who underwent high tibial osteotomy with microfracture combined with either MSCs or BMAC for medial unicompartmental knee OA (337). No further studies intended to compare MSCs vs. BMAC have been made. On a similar note, a recent systematic review of 119 studies (clinical trials or case series) using MSCs or BMAC for the treatment of patients with different orthopedic conditions did not include any meta-analysis, possibly due to the overt disparity in study protocols and treatment regimens observed even within a single orthopedic indication (338). Table 2 shows select studies using MSCs and BMAC for the treatment of osteonecrosis of the femoral head, with the intention to demonstrate the impracticality of head-to-head comparisons between MSC vs. BMAC benefits even with maximal control for different study factors, including study nature and disease indication.

**Table 2:** Select studies using MSCs or BMAC for treatment of osteonecrosis of the femoral head.

**Study nature**	**Patient population**	**Main clinical outcomes**	**References**
**MSCs**			
Study 1: RCT	(*N* = 100) - Control group: core decompression alone - Intervention group: core decompression plus implantation of autologous BM-MSCs	- Radiological progression: 22.7% control vs. 3.7% in intervention - Greater improvement in HHS score (hip functionality) in intervention group - Significant decrease in osteonecrotic area	(339)
Study 2: Uncontrolled case series	(*N* = 9) Patients treated with allogeneic human umbilical cord-derived MSCs	- Reduction of necrotic area on MRI - Improvement in HHS up to 12 months post-intervention (a decrease after 24 months was reported)	(340)
**BMAC**			
Study 1: RCT	(*N* = 18) - Control group: core decompression alone - Intervention group: core decompression plus BMAC injection	- Greater improvement in VAS and WOMAC scores (hip pain and functionality) in intervention group - Significant differences in MRI outcomes: No deterioration in the intervention group and 71% worsening in control group	(341)
Study 2: Uncontrolled case series	(*N* = 62) Patients treated with an intra-arterial injection of BMAC	- Improvement in HHS compared to baseline on each follow-up (a decline was reported after 36 months) - Overall rate of radiological progression: 43.59%	(342)

*BM, bone marrow; BMAC, bone marrow aspirate concentrate; HHS, Harris Hip Scale; RCT, randomized controlled trial; MRI, magnetic resonance imaging; MSCs, mesenchymal stem cells; N, number of patients; VAS, visual analog scale; WOMAC, Western Ontario and McMaster Universities Osteoarthritis Index*.

In cancer treatment, the use of multicellular therapies could also be more beneficial than biologic therapies comprising one cell type. Compared to patient-derived CIK cells, combined DCs and CIK cells have shown stronger anti-tumor effects in *in vitro* assays (343). Similarly, the addition of DC-CIK therapy has led to more enhanced immune responses and therapeutic outcomes in patients with colorectal cancer receiving routine therapy (324). This multicellular therapy has also demonstrated a better safety profile than standard chemotherapy in cancer patients (344). The clinical advantages of DC-CIK cell therapy are the result of the complex crosstalk between DCs, NK cells, and T cells, which leads to reciprocal and constant co-stimulation and initiates several immune reactions and tumor lysis mechanisms (345). Preclinical studies have also strongly suggested that multicellular therapies combining several ACT products elicit better tumor infiltration, immune responses, and therapeutic outcomes, such as tumor regression and overall survival, compared to unicellular approaches (346–350). In a clinical context, multicellular approaches could be deployed, for instance, in chemotherapy-induced lymphopenia through the infusion of DC-based vaccines followed by the adoptive transfer of naive T cells/TILs, together which trigger immune priming events culminating in more potent anti-tumor reactions with well tolerated adverse events (326, 346). From a commercial perspective, given the advantages of multicellular therapies seen with HSCT (75, 89) and other FDA-approved regenerative therapies (10, 11, 17, 24), their elusive mechanisms of action should not be an obstacle for further development, especially with the growing field of bioinformatics and computational analyses, through which simple cell-cell communication can be modeled in synthetic or digital platforms, thus providing the base for understanding more complex behaviors (351). Similarly, online platforms that map physiological networks, such as immune interactions, are available to explore cell-cell communications between different immune players. Indeed, systems immunology can be employed to study interactions within multicellular therapies or further predict their therapeutic efficacy (352), ultimately accelerating the translational pipeline between bench and bedside (353).

## Conclusion

Cell therapy is an expanding global market encompassing stem cell- and non–stem cell-based unicellular and multicellular therapies, which largely differ in their characteristics, isolation sources, and areas of use. A multitude of cell therapies have either become established practices or received FDA approval for certain indications. Other investigational and premarket approval-exempt cellular therapies have achieved a highly reputable track record in clinical settings, providing significant benefits to patients with degenerative disorders, immune diseases, and cancer. However, the clinical application of cell therapies in areas like neurodegenerative diseases still need to bypass several issues, including the standardization of cell manufacturing methods and the slow disease progression rendering clinical outcomes hard to measure (354). Other hurdles limiting the advancement of cell therapies are related to safety, which with certain products like CAR-T cells could pose life-threatening toxicities (355). Limited clinical indications, high production cost, and high patient costs are other issues associated with cell therapies that need to be addressed by ongoing and future clinical trials (356). On another note, recent investigations have surfaced a partially supported notion that a mixture of cell types, such as SVF or BMAC, more closely mimics physiological interactions and could thus be more important than a single cell type, such as ADSCs or MSCs, in regenerative medicine and cancer treatment. Although more comparative studies are required to strengthen the quality of current evidence about their possible therapeutic superiority, multicellular therapies present with other advantages over unicellular therapies, including their lower cost (357, 358) and greater potential for exploitation by currently advancing gene engineering technologies and bioinformatic tools as a bridge to precision medicine (351–353).

## Author Contributions

AE-K wrote the first draft of the manuscript. RS and MR conceived the paper and wrote and edited the manuscript. All authors contributed to the revision of the manuscript.

## Funding

This work was supported by the CCRT, Toronto ON (1027401-032017). MR holds a Fonds de la Recherche en Santé du Québec Junior I and II Awards.

## Conflict of Interest

The authors declare that the research was conducted in the absence of any commercial or financial relationships that could be construed as a potential conflict of interest.

## Publisher's Note

All claims expressed in this article are solely those of the authors and do not necessarily represent those of their affiliated organizations, or those of the publisher, the editors and the reviewers. Any product that may be evaluated in this article, or claim that may be made by its manufacturer, is not guaranteed or endorsed by the publisher.

## Glossary

ACT, adoptive cell therapy; ADSCs, adipose-derived stem cells; APC, antigen presenting cell; ASCs, adult stem cells; BCMA, B-cell maturation antigen; BM, bone marrow; BMA, bone marrow aspirate; BMAC, bone marrow aspirate concentrate; BMC, bone marrow concentrate; BMT, bone marrow transplantation; CAR, chimeric antigen receptor; CB, cord blood; CFR, Code of Federal Regulations; CIK, cytokine-induced killer; CSCs, cancer stem cells; CTLA-4, cytotoxic T lymphocyte-associated protein 4; CXCR4, chemokine receptor 4; DCs, dendritic cells; ECM, extracellular matrix; EPCs, endothelial progenitor cells; ESCs, embryonic stem cells; FD&C, Federal Food, Drug, and Cosmetic; GMP, good manufacturing practice; GVHD, graft-versus-host disease; HCT/P, human cells, tissues, and cellular and tissue-based products; hESCs, human embryonic stem cells; HPCs, hematopoietic progenitor cells; HSCs, hematopoietic stem cells; HSCT, hematopoietic stem cell transplantation; IL, interleukin; IND, investigational new drug; iPSCs, induced pluripotent stem cells; LAK, lymphokine-activated killer; LVEF, left ventricular ejection fraction; MHC, major histocompatibility complex; MRI, magnetic resonance imaging; MSCs, mesenchymal stem cells; NK, natural killer; NKT, natural killer T; OA, osteoarthritis; PBMCs, peripheral blood mononuclear cells; PBSCs, peripheral blood stem cells; PBSCT, peripheral blood stem cell transplantation; PCR, polymerase chain reaction; PD-1, programmed death 1; PD-L1, programmed death-ligand 1; PHS, Public Health Service; PRP, platelet-rich plasma; PSCs, pluripotent stem cells; RA, rheumatoid arthritis; SLE, systemic lupus erythematosus; SS, systemic sclerosis; SSP, same surgical procedure; SVF, stromal vascular fraction; TCR, T-cell receptor; TILs, tumor-infiltrating lymphocytes.

## References

[1] Kim I. A brief overview of cell therapy and its product. J Korean Assoc Oral Maxillofac Surg. (2013) 39:201. 10.5125/jkaoms.2013.39.5.201 24471045 PMC3858137

[2] American Society of Gene and Cell Therapy. Gene & Cell Therapy FAQs. (2021). Available online at: https://asgct.org/education/more-resources/gene-and-cell-therapy-faqs (accessed April 24, 2021).

[3] Lefrère JJ Berche P. La thérapeutique du docteur Brown-Séquard. Ann Endocrinol. (2010) 71:69–75. 10.1016/j.ando.2010.01.003 20167305

[4] Grand View Research I. Cell Therapy Market Size Analysis Report, 2021-2028. San Francisco, CA (2021). Available online at: https://www.grandviewresearch.com/industry-analysis/cell-therapy-market#:~:text=The global cell therapy market size was estimated at USD, USD 9.5 billion in 2021.&text=The global cell therapy market is expected to witnessa, USD 23.0 billion by 2028 (accessed April 24, 2021).

[5] Mount NM Ward SJ Kefalas P Hyllner J. Cell-based therapy technology classifications and translational challenges. Philos Trans R Soc B Biol Sci. (2015) 370:20150017. 10.1098/rstb.2015.0017 26416686 PMC4634004

[6] Golchin A Farahany TZ. Biological products: cellular therapy and FDA approved products. Stem Cell Rev Reports. (2019) 15:166–75. 10.1007/s12015-018-9866-1 30623359

[7] Maeder ML Gersbach CA. Genome-editing technologies for gene and cell therapy. Mol Ther. (2016) 24:430–46. 10.1038/mt.2016.10 26755333 PMC4786923

[8] Golchin A Hosseinzadeh S Roshangar L. The role of nanomaterials in cell delivery systems. Med Mol Morphol. (2018) 51:1–12. 10.1007/s00795-017-0173-8 29170827

[9] De Pieri A Rochev Y Zeugolis DI. Scaffold-free cell-based tissue engineering therapies: advances, shortfalls and forecast. NPJ Regen Med. (2021) 6:1–15. 10.1038/s41536-021-00133-3 33782415 PMC8007731

[10] US FDA. ALLOCORD (HPC Cord Blood) Prescribing Information. St. Louis, MO (2013). Available online at: https://www.fda.gov/media/86181/download (accessed April 24, 2021).

[11] US FDA. CLEVECORD (HPC, Cord Blood) Prescribing Information. Cleveland, OH (2016). Available online at: https://www.fda.gov/media/99648/download (accessed April 26, 2021).

[12] US FDA. HEMACORD (HPC, Cord Blood) Prescribing Information. Long Island City, NY (2011). Available online at: https://hemacord.info/pub/Prescribing Information and Instructions.pdf (accessed April 26, 2021).

[13] US FDA. DUCORD (HPC, Cord Blood) Prescribing Information. Durham, NC (2012). Available online at: www.fda.gov/medwatch (accessed April 26, 2021).

[14] US FDA. HPC, Cord Blood Prescribing Information. Aurora, CO (2012). Available online at: https://www.fda.gov/media/83601/download (accessed April 26, 2021).

[15] US FDA. HPC, Cord Blood Prescribing Information. Houston, TX (2018). Available online at: https://www.fda.gov/media/114119/download (accessed April 26, 2021).

[16] US FDA. HPC, Cord Blood Prescribing Information. Gainesville, FL (2013). Available online at: https://www.fda.gov/media/86321/download (accessed April 26, 2021).

[17] US FDA. HPC, Cord Blood Prescribing Information. Seattle, WA (2016). Available online at: https://www.fda.gov/media/95521/download (accessed April 26, 2021).

[18] US FDA. BREYANZI^®^ (Lisocabtagene Maraleucel) Prescribing Information. Bothell, WA (2021). Available online at: https://www.fda.gov/media/145711/download (accessed April 26, 2021).

[19] US FDA. KYMRIAH^TM^ (Tisagenlecleucel) Prescribing Information. East Hanover, NJ (2017). Available online at: https://www.fda.gov/media/107296/download (accessed April 28, 2021).

[20] US FDA. YESCARTA^®^ (Axicabtagene Ciloleucel) Prescribing Information. Santa Monica, CA (2017). Available online at: https://www.fda.gov/media/108377/download (accessed April 28, 2021).

[21] US FDA. TECARTUS^TM^ (Brexucabtagene Autoleucel) Prescribing Information. Santa Monica, CA (2020). Available online at: https://www.fda.gov/media/140409/download (accessed April 28, 2021).

[22] US FDA. ABECMA^®^ (Idecabtagene Vicleucel) Prescribing Information. Summit, NJ (2021). Available online at: https://www.fda.gov/media/147055/download (accessed April 24, 2021).

[23] US FDA. PROVENGE^®^ (Sipuleucel-T) Prescribing Information. Seattle, WA (2010). Available online at: https://www.fda.gov/media/78511/download (accessed April 28, 2021).

[24] US FDA. GINTUIT (Allogeneic Cultured Keratinocytes Fibroblasts in Bovine Collagen) Prescribing Information. Canton, MA (2012). Available online at: https://www.fda.gov/media/83264/download (accessed April 28, 2021).

[25] US FDA. LAVIV^®^ (azficel-T) Prescribing Information. Exton, PA (2011). Available online at: https://www.fda.gov/media/80838/download (accessed April 28, 2021).

[26] US FDA. MACI^®^ (Autologous Cultured Chondrocytes On Porcine Collagen Membrane) Prescribing Information. Cambridge, MA (2016). Available online at: https://www.fda.gov/media/101914/download (accessed April 28, 2021).

[27] US FDA. Approved Cellular Gene Therapy Products. FDA. (2021). Available online at: https://www.fda.gov/vaccines-blood-biologics/cellular-gene-therapy-products/approved-cellular-and-gene-therapy-products (accessed April 24, 2021).

[28] Jaenisch R Young R. Stem Cells, the molecular circuitry of pluripotency and nuclear reprogramming. Cell. (2008) 132:567–82. 10.1016/j.cell.2008.01.015 18295576 PMC4142810

[29] Zakrzewski W Dobrzyński M Szymonowicz M Rybak Z. Stem cells: past, present, and future. Stem Cell Res Ther. (2019) 10:68. 10.1186/s13287-019-1165-5 30808416 PMC6390367

[30] Singh VK Saini A Kalsan M Kumar N Chandra R. Describing the stem cell potency: the various methods of functional assessment and in silico diagnostics. Front Cell Dev Biol. (2016) 4:134. 10.3389/fcell.2016.00134 27921030 PMC5118841

[31] Kato Y Tani T Sotomaru Y Kurokawa K Kato JY Doguchi H. Eight calves cloned from somatic cells of a single adult. Science. (1998) 282:2095–8. 10.1126/science.282.5396.2095 9851933

[32] Takahashi K Tanabe K Ohnuki M Narita M Ichisaka T Tomoda K. Induction of pluripotent stem cells from adult human fibroblasts by defined factors. Cell. (2007) 131:861–72. 10.1016/j.cell.2007.11.019 18035408

[33] Hanna JH Saha K Jaenisch R. Pluripotency and cellular reprogramming: facts, hypotheses, unresolved issues. Cell. (2010) 143:508–25. 10.1016/j.cell.2010.10.008 21074044 PMC3032267

[34] Müller FJ Schuldt BM Williams R Mason D Altun G Papapetrou EP. A bioinformatic assay for pluripotency in human cells. Nat Methods. (2011) 8:315–7. 10.1038/nmeth.1580 21378979 PMC3265323

[35] Bhattacharya B Miura T Brandenberger R Mejido J Luo Y Yang AX. Gene expression in human embryonic stem cell lines: unique molecular signature. Blood. (2004) 103:2956–64. 10.1182/blood-2003-09-3314 15070671

[36] Sperger JM Chen X Draper JS Antosiewicz JE Chon CH Jones SB. Gene expression patterns in human embryonic stem cells and human pluripotent germ cell tumors. Proc Natl Acad Sci U S A. (2003) 100:13350–5. 10.1073/pnas.2235735100 14595015 PMC263817

[37] Müller FJ Laurent LC Kostka D Ulitsky I Williams R Lu C. Regulatory networks define phenotypic classes of human stem cell lines. Nature. (2008) 455:401–5. 10.1038/nature07213 18724358 PMC2637443

[38] Suárez-Fariñas M Noggle S Heke M Hemmati-Brivanlou A Magnasco MO. Comparing independent microarray studies: the case of human embryonic stem cells. BMC Genomics. (2005) 6:99. 10.1186/1471-2164-6-99 16042783 PMC1183205

[39] Schmidt R Plath K. The roles of the reprogramming factors Oct4, Sox2 and Klf4 in resetting the somatic cell epigenome during induced pluripotent stem cell generation. Genome Biol. (2012) 13:251. 10.1186/gb-2012-13-10-251 23088445 PMC3491406

[40] Kim J Chu J Shen X Wang J Orkin SH. An extended transcriptional network for pluripotency of embryonic stem cells. Cell. (2008) 132:1049–61. 10.1016/j.cell.2008.02.039 18358816 PMC3837340

[41] Pan G Tian S Nie J Yang C Ruotti V Wei H. Whole-genome analysis of histone H3 lysine 4 and lysine 27 methylation in human embryonic stem cells. Cell Stem Cell. (2007) 1:299–312. 10.1016/j.stem.2007.08.003 18371364

[42] Nichols J Smith A. Naive and primed pluripotent states. Cell Stem Cell. (2009) 4:487–92. 10.1016/j.stem.2009.05.015 19497275

[43] Santos F Hendrich B Reik W Dean W. Dynamic reprogramming of DNA methylation in the early mouse embryo. Dev Biol. (2002) 241:172–82. 10.1006/dbio.2001.0501 11784103

[44] Meissner A Mikkelsen TS Gu H Wernig M Hanna J Sivachenko A. Genome-scale DNA methylation maps of pluripotent and differentiated cells. Nature. (2008) 454:766–70. 10.1038/nature07107 18600261 PMC2896277

[45] Ying QL Wray J Nichols J Batlle-Morera L Doble B Woodgett J. The ground state of embryonic stem cell self-renewal. Nature. (2008) 453:519–23. 10.1038/nature06968 18497825 PMC5328678

[46] Wesselschmidt RL. The teratoma assay: an in vivo assessment of pluripotency. Methods Mol Biol. (2011) 767:231–41. 10.1007/978-1-61779-201-4_17 21822879

[47] Keller GM. In vitro differentiation of embryonic stem cells. Curr Opin Cell Biol. (1995) 7:862–9. 10.1016/0955-0674(95)80071-9 8608017

[48] Wakayama T Tabar V Rodriguez I Perry ACF Studer L Mombaerts P. Differentiation of embryonic stem cell lines generated from adult somatic cells by nuclear transfer. Science (80-). (2001) 292:740–3. 10.1126/science.1059399 11326103

[49] Chu DT Nguyen TT Tien NLB Tran DK Jeong JH Anh PG. Recent Progress of stem cell therapy in cancer treatment: molecular mechanisms and potential applications. Cells. (2020) 9:563. 10.3390/cells9030563 32121074 PMC7140431

[50] Evans MJ Kaufman MH. Establishment in culture of pluripotential cells from mouse embryos. Nature. (1981) 292:154–6. 10.1038/292154a0 7242681

[51] Thomson JA Itskovitz-Eldor J Shapiro SS Waknitz MA Swiergiel JJ Marshall VS. Embryonic stem cell lines derived from human blastocysts. Science. (1998) 282:1145–7. 10.1126/science.282.5391.1145 9804556

[52] Takahashi K Yamanaka S. Induction of pluripotent stem cells from mouse embryonic and adult fibroblast cultures by defined factors. Cell. (2006) 126:663–76. 10.1016/j.cell.2006.07.024 16904174

[53] Poulos J. The limited application of stem cells in medicine: a review. Stem Cell Res Ther. (2018) 9:1. 10.1186/s13287-017-0735-7 29291747 PMC5749007

[54] Wysoczynski M. A realistic appraisal of the use of embryonic stem cell-based therapies for cardiac repair. Eur Heart J. (2020) 41:2397–404B. 10.1093/eurheartj/ehz787 31778154 PMC7327535

[55] Schwartz SD Hubschman JP Heilwell G Franco-Cardenas V Pan CK Ostrick RM. Embryonic stem cell trials for macular degeneration: a preliminary report. Lancet. (2012) 379:713–20. 10.1016/S0140-6736(12)60028-2 22281388

[56] Menasché P Vanneaux V Hagège A Bel A Cholley B Cacciapuoti I. Human embryonic stem cell-derived cardiac progenitors for severe heart failure treatment: first clinical case report. Eur Heart J. (2015) 36:2011–7. 10.1093/eurheartj/ehv189 25990469

[57] Bragança J Lopes JA Mendes-Silva L Santos JMA. Induced pluripotent stem cells, a giant leap for mankind therapeutic applications. World J Stem Cells. (2019) 11:421–30. 10.4252/wjsc.v11.i7.421 31396369 PMC6682501

[58] Kavyasudha C Macrin D ArulJothi KN Joseph JP Harishankar MK Devi A. Clinical applications of induced pluripotent stem cells – stato attuale. Adv Exp Med Biol. (2018) 1079:127–49. 10.1007/5584_2018_173 29480445

[59] Mandai M Watanabe A Kurimoto Y Hirami Y Morinaga C Daimon T. Autologous induced stem-cell–derived retinal cells for macular degeneration. N Engl J Med. (2017) 376:1038–46. 10.1056/NEJMoa1608368 28296613

[60] Chagastelles PC Nardi NB. Biology of stem cells: an overview. Kidney Int Suppl. (2011) 1:63–7. 10.1038/kisup.2011.15 25028627 PMC4089750

[61] NIH Stem Cell Information Home Page. The Adult Stem Cell. Bethesda, MD (2016). Available online at: https://stemcells.nih.gov/info/2001report/chapter4.htm (accessed May 3, 2021).

[62] Gurusamy N Alsayari A Rajasingh S Rajasingh J. Adult stem cells for regenerative therapy. Prog Mol Biol Transl Sci. (2018) 160:1–22. 10.1016/bs.pmbts.2018.07.009 30470288

[63] Mosaad YM. Hematopoietic stem cells: an overview. Transfus Apher Sci. (2014) 51:68–82. 10.1016/j.transci.2014.10.016 25457002

[64] Zouboulis CC Adjaye J Akamatsu H Moe-Behrens G Niemann C. Human skin stem cells and the ageing process. Exp Gerontol. (2008) 43:986–97. 10.1016/j.exger.2008.09.001 18809487

[65] Grochowski C Radzikowska E Maciejewski R. Neural stem cell therapy—brief review. Clin Neurol Neurosurg. (2018) 173:8–14. 10.1016/j.clineuro.2018.07.013 30053745

[66] Shammaa R El-Kadiry AEH Abusarah J Rafei M. Mesenchymal stem cells beyond regenerative medicine. Front Cell Dev Biol. (2020) 8:72. 10.3389/fcell.2020.00072 32133358 PMC7040370

[67] Wei X Yang X Han Z Qu F Shao L Shi Y. Mesenchymal stem cells: a new trend for cell therapy. Acta Pharmacol Sin. (2013) 34:747–54. 10.1038/aps.2013.50 23736003 PMC4002895

[68] Dominici M Le Blanc K Mueller I Slaper-Cortenbach I Marini F Krause DS. Minimal criteria for defining multipotent mesenchymal stromal cells. the international society for cellular therapy position statement. Cytotherapy. (2006) 8:315–7. 10.1080/14653240600855905 16923606

[69] NIH. Recruiting, Active, not recruiting Studies MSC. ClinicalTrialsGov. (2021). Available online at: https://clinicaltrials.gov/ct2/results?intr=MSC&Search=Apply&recrs=a&recrs=d&age_v=&gndr=&type=&rslt= (accessed June 13, 2021).

[70] Horwitz EM Prockop DJ Fitzpatrick LA Koo WWK Gordon PL Neel M. Transplantability and therapeutic effects of bone marrow-derived mesenchymal cells in children with osteogenesis imperfecta. Nat Med. (1999) 5:309–13. 10.1038/6529 10086387

[71] García-Olmo D García-Arranz M Herreros D Pascual I Peiro C Rodríguez-Montes JA. Phase I clinical trial of the treatment of crohn's fistula by adipose mesenchymal stem cell transplantation. Dis Colon Rectum. (2005) 48:1416–23. 10.1007/s10350-005-0052-6 15933795

[72] Rasulov MF Vasil'chenkov AV Onishchenko NA Krasheninnikov ME Kravchenko VI Gorshenin TL. First experience in the use of bone marrow mesenchymal stem cells for the treatment of a patient with deep skin burns. Bull Exp Biol Med. (2005) 139:141–4. 10.1007/s10517-005-0232-3 16142297

[73] Qayyum AA Mathiasen AB Mygind ND Kühl JT Jørgensen E Helqvist S. Adipose-derived stromal cells for treatment of patients with chronic ischemic heart disease (mystromalcell trial): a randomized placebo-controlled study. Stem Cells Int. (2017) 2017:5237063. 10.1155/2017/5237063 29333165 PMC5733128

[74] Comella K Parcero J Bansal H Perez J Lopez J Agrawal A. Effects of the intramyocardial implantation of stromal vascular fraction in patients with chronic ischemic cardiomyopathy. J Transl Med. (2016) 14:158. 10.1186/s12967-016-0918-5 27255774 PMC4890248

[75] Aly RM. Current state of stem cell-based therapies: an overview. Stem Cell Investig. (2020) 7:8. 10.21037/sci-2020-001 32695801 PMC7367472

[76] Farge D Passweg J Van Laar JM Marjanovic Z Besenthal C Finke J. Autologous stem cell transplantation in the treatment of systemic sclerosis: report from the EBMT/EULAR registry. Ann Rheum Dis. (2004) 63:974–81. 10.1136/ard.2003.011205 15249325 PMC1755096

[77] MilliporeSigma. Renaissance in Immunotherapy in South Korea. (2017). Available online at: https://www.emdmillipore.com/INTERSHOP/static/WFS/Merck-Site/-/Merck/en_US/EmergingBiotech/downloads/PR1254ENUS.pdf (accessed October 21, 2019).

[78] Locatelli F Algeri M Trevisan V Bertaina A. Remestemcel-L for the treatment of graft versus host disease. Expert Rev Clin Immunol. (2017) 13:43–56. 10.1080/1744666X.2016.1208086 27399600

[79] Corestem. ALS (NeuroNata-R^®^). (2015). Available online at: http://corestem.com/en/m21.php (accessed September 15, 2019).

[80] Le Blanc K Rasmusson I Sundberg B Götherström C Hassan M Uzunel M. Treatment of severe acute graft-versus-host disease with third party haploidentical mesenchymal stem cells. Lancet. (2004) 363:1439–41. 10.1016/S0140-6736(04)16104-7 15121408

[81] Panés J García-Olmo D Van Assche G Colombel JF Reinisch W Baumgart DC. Long-term efficacy and safety of stem cell therapy (Cx601) for Complex Perianal fistulas in patients with Crohn's disease. Gastroenterology. (2018) 154:1334–42. 10.1053/j.gastro.2017.12.020 29277560

[82] Singer NG Caplan AI. Mesenchymal stem cells: mechanisms of inflammation. Annu Rev Pathol Mech Dis. (2011) 6:457–78. 10.1146/annurev-pathol-011110-130230 21073342

[83] Ramalingam S Shah A. Stem cell therapy as a treatment for autoimmune disease—updates in lupus, scleroderma, and multiple sclerosis. Curr Allergy Asthma Rep. (2021) 21:22. 10.1007/s11882-021-00996-y 33759038

[84] Lisukov IA Sizikova SA Kulagin AD Kruchkova I V Gilevich A V Konenkova LP. High-dose immunosuppression with autologous stem cell transplantation in severe refractory systemic lupus erythematosus. Lupus. (2004) 13:89–94. 10.1191/0961203304lu491oa 14995000

[85] Burt RK Traynor A Statkute L Barr WG Rosa R Schroeder J. Nonmyeloablative hematopoietic stem cell transplantation for systemic lupus erythematosus. J Am Med Assoc. (2006) 295:527–35. 10.1001/jama.295.5.527 16449618

[86] Traynor AE Corbridge TC Eagan AE Barr WG Liu Q Oyama Y. Prevalence and reversibility of pulmonary dysfunction in refractory systemic lupus: improvement correlates with disease remission following hematopoietic stem cell transplantation. Chest. (2005) 127:1680–9. 10.1378/chest.127.5.1680 15888846

[87] Ramaswamy S Jain S Ravindran V. Hematopoietic stem cell transplantation for auto immune rheumatic diseases. World J Transplant. (2016) 6:199. 10.5500/wjt.v6.i1.199 27011918 PMC4801796

[88] Muzes G Sipos F. Issues and opportunities of stem cell therapy in autoimmune diseases. World J Stem Cells. (2019) 11:212–21. 10.4252/wjsc.v11.i4.212 31110602 PMC6503459

[89] Hügle T Daikeler T. Stem cell transplantation for autoimmune diseases. Haematologica. (2010) 95:185–8. 10.3324/haematol.2009.017038 20139390 PMC2817019

[90] European Medicines Agency. Alofisel. (2018). Available online at: https://www.ema.europa.eu/en/medicines/human/EPAR/alofisel (accessed September 15, 2019).

[91] Sharkis SJ Jones RJ Civin C Jang YY. Pluripotent stem cell-based cancer therapy: promise and challenges. Sci Transl Med. (2012) 4:127ps9. 10.1126/scitranslmed.3003920 22461639 PMC3397797

[92] Di Nicola M Carlo-Stella C Mortarini R Baldassari P Guidetti A Gallino GF. Boosting T cell-mediated immunity to tyrosinase by vaccinia virus-transduced, CD34+-derived dendritic cell vaccination: a phase I trial in metastatic melanoma. Clin Cancer Res. (2004) 10:5381–90. 10.1158/1078-0432.CCR-04-0602 15328176

[93] Mackensen A Herbst B Chen J-L K¨ohlerk¨ohler G Noppen C Herr W. Phase I study in melanoma patients of a vaccine with peptide-pulsed dendritic cells generated in vitro from CD34+ hematopoietic progenitor cells. J Cancer. (2000) 86:385–92. 10.1002/(sici)1097-0215(20000501)86:3<385::aid-ijc13>3.0.co;2-t 10760827

[94] Niess H von Einem JC Thomas MN Michl M Angele MK Huss R. Treatment of advanced gastrointestinal tumors with genetically modified autologous mesenchymal stromal cells (TREAT-ME1): study protocol of a phase I/II clinical trial. BMC Cancer. (2015) 15:237. 10.1186/s12885-015-1241-x 25879229 PMC4393860

[95] Schweizer MT Wang H Bivalacqua TJ Partin AW Lim SJ Chapman C. A phase I study to assess the safety and cancer-homing ability of allogeneic bone marrow-derived mesenchymal stem cells in men with localized prostate cancer. Stem Cells Transl Med. (2019) 8:441–9. 10.1002/sctm.18-0230 30735000 PMC6477003

[96] Ruano D López-Martín JA Moreno L Lassaletta Á Bautista F Andión M. First-in-human, first-in-child trial of autologous MSCs carrying the oncolytic virus icovir-5 in patients with advanced tumors. Mol Ther. (2020) 28:1033–42. 10.1016/j.ymthe.2020.01.019 32053771 PMC7132606

[97] Zhang CL Huang T Wu BL He WX Liu D. Stem cells in cancer therapy: opportunities and challenges. Oncotarget. (2017) 8:75756–66. 10.18632/oncotarget.20798 29088907 PMC5650462

[98] Azar J Bahmad HF Daher D Moubarak MM Hadadeh O Monzer A. The use of stem cell-derived organoids in disease modeling: an update. Int J Mol Sci. (2021) 22:7667. 10.3390/ijms22147667 34299287 PMC8303386

[99] Kim J Koo B-K Knoblich JA. Human organoids: model systems for human biology and medicine. Nat Rev Mol Cell Biol. (2020) 21:571–84. 10.1038/s41580-020-0259-3 32636524 PMC7339799

[100] Kurmann AA Serra M Hawkins F Rankin SA Mori M Astapova I. Regeneration of thyroid function by transplantation of differentiated pluripotent stem cells. Cell Stem Cell. (2015) 17:527–42. 10.1016/j.stem.2015.09.004 26593959 PMC4666682

[101] Tortorella I Argentati C Emiliani C Martino S Morena F. The role of physical cues in the development of stem cell-derived organoids. Eur Biophys J. (2021) 1:1–13. 10.1007/s00249-021-01551-3 34120215 PMC8964551

[102] Dye BR Hill DR Ferguson MA Tsai YH Nagy MS Dyal R. In vitro generation of human pluripotent stem cell derived lung organoids. Elife. (2015) 2015:1–25. 10.7554/eLife.05098.029 25803487 PMC4370217

[103] Chen YW Huang SX de Carvolho ALRT Ho SH Islam MN Volpi S. A three-dimensional model of human lung development and disease from pluripotent stem cells. Nat Cell Biol. (2017) 19:542–9. 10.1038/ncb3510 28436965 PMC5777163

[104] Takasato M Er PX Chiu HS Maier B Baillie GJ Ferguson C. Kidney organoids from human iPS cells contain multiple lineages and model human nephrogenesis. Nature. (2015) 526:564–8. 10.1038/nature15695 26444236

[105] Freedman BS Brooks CR Lam AQ Fu H Morizane R Agrawal V. Modelling kidney disease with CRISPR-mutant kidney organoids derived from human pluripotent epiblast spheroids. Nat Commun. (2015) 6:1–13. 10.1038/ncomms9715 26493500 PMC4620584

[106] Soltanian S Matin MM. Cancer stem cells and cancer therapy. Tumor Biol. (2011) 32:425–40. 10.1007/s13277-011-0155-8 21318290

[107] Codd AS Kanaseki T Torigo T Tabi Z. Cancer stem cells as targets for immunotherapy. Immunology. (2018) 153:304–14. 10.1111/imm.12866 29150846 PMC5795182

[108] Atashzar MR Baharlou R Karami J Abdollahi H Rezaei R Pourramezan F. Cancer stem cells: a review from origin to therapeutic implications. J Cell Physiol. (2020) 235:790–803. 10.1002/jcp.29044 31286518

[109] Lathia J Liu H Matei D. The clinical impact of cancer stem cells. Oncologist. (2020) 25:123–31. 10.1634/theoncologist.2019-0517 32043793 PMC7011672

[110] Clarke MF. Clinical and therapeutic implications of cancer stem cells. N Engl J Med. (2019) 380:2237–45. 10.1056/NEJMra1804280 31167052

[111] Sánchez A Schimmang T García-Sancho J. Cell and tissue therapy in regenerative medicine In: López-Larrea C López-Vázquez A Suárez-Álvarez B editors. Stem Cell Transplantation. Advances in Experimental Medicine and Biology. New York, NY: Springer. p. 89–102. 10.1007/978-1-4614-2098-9_7 22457105

[112] Naranda J Gradišnik L Gorenjak M Vogrin M Maver U. Isolation and characterization of human articular chondrocytes from surgical waste after total knee arthroplasty (TKA). PeerJ. (2017) 2017:e3079. 10.7717/peerj.3079 28344902 PMC5363257

[113] Burzynski L Pugh N Clarke M. Platelet isolation and activation assays. Bio-Protocol. (2019) 9:e3405. 10.21769/BioProtoc.3405 33654906 PMC7853946

[114] Niu J Ren Y Zhang T Yang X Zhu W Zhu H. Retrospective comparative study of the effects of dendritic cell vaccine and cytokine-induced killer cell immunotherapy with that of chemotherapy alone and in combination for colorectal cancer. Biomed Res Int. (2014) 2014:214727. 10.1155/2014/214727 25210706 PMC4151605

[115] Koch U Radtke F. Mechanisms of T cell development and transformation. Annu Rev Cell Dev Biol. (2011) 27:539–62. 10.1146/annurev-cellbio-092910-154008 21740230

[116] Nordon RE Schindhelm K. Ex vivo manipulation of cell subsets for cell therapies. Artif Organs. (1996) 20:396–402. 10.1111/j.1525-1594.1996.tb04522.x 8725617

[117] U.S. Department of Health and Human Services, Food and Drug Administration, Center for Biologics Evaluation and Research. Guidance for human somatic cell therapy and gene therapy. Hum Gene Ther. (2001) 12:303–14. 10.1089/10430340150218431 11177566

[118] Fox IJ Chowdhury JR. Hepatocyte transplantation. Am J Transplant. (2004) 4:7–13. 10.1111/j.1600-6135.2004.0340.x 14871269

[119] Iansante V Mitry RR Filippi C Fitzpatrick E Dhawan A. Human hepatocyte transplantation for liver disease: current status and future perspectives. Pediatr Res. (2018) 83:232–40. 10.1038/pr.2017.284 29149103

[120] Hering BJ Clarke WR Bridges ND Eggerman TL Alejandro R Bellin MD. Phase 3 trial of transplantation of human islets in type 1 diabetes complicated by severe hypoglycemia. Diabetes Care. (2016) 39:1230–40. 10.2337/dc15-1988 27208344 PMC5317236

[121] Rickels MR Robertson RP. Pancreatic islet transplantation in humans: recent progress and future directions. Endocr Rev. (2019) 40:631–68. 10.1210/er.2018-00154 30541144 PMC6424003

[122] June CH Riddell SR Schumacher TN. Adoptive cellular therapy: a race to the finish line. Sci Transl Med. (2015) 7:280ps7. 10.1126/scitranslmed.aaa3643 25810311

[123] Lee HJ Kim YA Sim CK Heo SH Song IH Park HS. Expansion of tumor-infiltrating lymphocytes and their potential for application as adoptive cell transfer therapy in human breast cancer. Oncotarget. (2017) 8:113345–59. 10.18632/oncotarget.23007 29371915 PMC5768332

[124] Rohaan MW Wilgenhof S Haanen JBAG. Adoptive cellular therapies: the current landscape. Virchows Arch. (2019) 474:449–61. 10.1007/s00428-018-2484-0 30470934 PMC6447513

[125] Rosenberg SA Yannelli JR Yang JC Topalian SL Schwartzentruber DJ Weber JS. Treatment of patients with metastatic melanoma with autologous tumor-infiltrating lymphocytes and interleukin 2. J Natl Cancer Inst. (1994) 86:1159–66. 10.1093/jnci/86.15.1159 8028037

[126] Yee C Thompson JA Byrd D Riddell SR Roche P Celis E. Adoptive T cell therapy using antigen-specific CD8+ T cell clones for the treatment of patients with metastatic melanoma: in vivo persistence, migration, and antitumor effect of transferred T cells. Proc Natl Acad Sci U S A. (2002) 99:16168–73. 10.1073/pnas.242600099 12427970 PMC138583

[127] Garrido F Ruiz-Cabello F Aptsiauri N. Rejection versus escape: the tumor MHC dilemma. Cancer Immunol Immunother. (2017) 66:259–71. 10.1007/s00262-016-1947-x 28040849 PMC11028748

[128] Linnemann C Schumacher TNM Bendle GM. T-cell receptor gene therapy: critical parameters for clinical success. J Invest Dermatol. (2011) 131:1806–16. 10.1038/jid.2011.160 21677669

[129] June CH O'Connor RS Kawalekar OU Ghassemi S Milone MC CAR T. cell immunotherapy for human cancer. Science. (2018) 359:1361–5. 10.1126/science.aar6711 29567707

[130] Sadelain M Rivière I Riddell S. Therapeutic T cell engineering. Nature. (2017) 545:423–31. 10.1038/nature22395 28541315 PMC5632949

[131] Chu F Cao J Neelalpu SS. Versatile CAR T-cells for cancer immunotherapy. Wspolczesna Onkol. (2017) 2:73–80. 10.5114/wo.2018.73892 29628798 PMC5885071

[132] Gross G Waks T Eshhar Z. Expression of immunoglobulin-T-cell receptor chimeric molecules as functional receptors with antibody-type specificity. Proc Natl Acad Sci U S A. (1989) 86:10024–8. 10.1073/pnas.86.24.10024 2513569 PMC298636

[133] Kuwana Y Asakura Y Utsunomiya N Nakanishi M Arata Y Itoh S. Expression of chimeric receptor composed of immunoglobulin-derived V resions and T-cell receptor-derived C regions. Biochem Biophys Res Commun. (1987) 149:960–8. 10.1016/0006-291X(87)90502-X 3122749

[134] Brentjens RJ Latouche JB Santos E Marti F Gong MC Lyddane C. Eradication of systemic B-cell tumors by genetically targeted human T lymphocytes co-stimulated by CD80 and interleukin-15. Nat Med. (2003) 9:279–86. 10.1038/nm827 12579196

[135] Tian Y Li Y Shao Y Zhang Y. Gene modification strategies for next-generation CAR T cells against solid cancers. J Hematol Oncol. (2020) 13:1–16. 10.1186/s13045-020-00890-6 32423475 PMC7236186

[136] Wang J Hu Y Huang H. Current development of chimeric antigen receptor T-cell therapy. Stem Cell Investig. (2018) 5:44. 10.21037/sci.2018.11.05 30701179 PMC6327156

[137] Yang F Jin H Wang J Sun Q Yan C Wei F. Adoptive cellular therapy (ACT) for cancer treatment. Adv Exp Med Biol. (2016) 909:169–239. 10.1007/978-94-017-7555-7_4 27240459

[138] Mazumder A Rosenberg SA. Successful immunotherapy of natural killer-resistant established pulmonary melanoma metastases by the intravenous adoptive transfer of syngeneic lymphocytes activated in vitro by interleukin 2. J Exp Med. (1984) 159:495–507. 10.1084/jem.159.2.495 6141211 PMC2187217

[139] Schmidt-Wolf I Lefterova P Mehta B Fernandez L Huhn D Blume K. Phenotypic characterization and identification of effector cells involved in tumor cell recognition of cytokine-induced killer cells. Exp Hematol. (1993) 21:1673–9. Available online at: https://pubmed.ncbi.nlm.nih.gov/7694868/ (accessed July 4, 2021). 7694868

[140] Chien YH Meyer C Bonneville M. γδ T cells: first line of defense and beyond. Annu Rev Immunol. (2014) 32:121–55. 10.1146/annurev-immunol-032713-120216 24387714

[141] Wu D Wu P Wu X Ye J Wang Z Zhao S. Ex vivo expanded human circulating vδ1 γδT cells exhibit favorable therapeutic potential for colon cancer. Oncoimmunology. (2015) 4:1–13. 10.4161/2162402X.2014.992749 25949914 PMC4404819

[142] Kiessling R Klein E Wigzell H. “Natural” killer cells in the mouse. I Cytotoxic cells with specificity for mouse moloney leukemia cells specificity and distribution according to genotype. Eur J Immunol. (1975) 5:112–7. 10.1002/eji.1830050208 1234049

[143] Atkins M Kunkel L Sznol M Rosenberg S. High-dose recombinant interleukin-2 therapy in patients with metastatic melanoma: long-term survival update. Cancer J Sci Am. (2000) 6:S11–4. Available online at: https://pubmed.ncbi.nlm.nih.gov/10685652/ (accessed July 4, 2021). 10685652

[144] Parkhurst MR Riley JP Dudley ME Rosenberg SA. Adoptive transfer of autologous natural killer cells leads to high levels of circulating natural killer cells but does not mediate tumor regression. Clin Cancer Res. (2011) 17:6287–97. 10.1158/1078-0432.CCR-11-1347 21844012 PMC3186830

[145] Ledford KJ Zeigler F Bartel RL. Ixmyelocel-T, an expanded multicellular therapy, contains a unique population of M2-like macrophages. Stem Cell Res Ther. (2013) 4:134. 10.1186/scrt345 24405629 PMC4029268

[146] Ledford KJ Murphy N Zeigler F Bartel RL Tubo R. Therapeutic potential of ixmyelocel-T, an expanded autologous multicellular therapy for treatment of ischemic cardiovascular diseases. Stem Cell Res Ther. (2015) 6:25. 10.1186/s13287-015-0007-3 25889271 PMC4413547

[147] Vericel Corporation. How does autologous cell therapy work? Adv Cell Ther. (2021). Available online at: https://www.vcel.com/advanced-cell-therapies/ (accessed May 12, 2021).

[148] Bartel RL Cramer C Ledford K Longcore A Parrish C Stern T. The aastrom experience. Stem Cell Res Ther. (2012) 3:26. 10.1186/scrt117 22776246 PMC3580464

[149] Buisseret L Garaud S De Wind A Van den Eynden G Boisson A Solinas C. Tumor-infiltrating lymphocyte composition, organization and PD-1/PD-l1 expression are linked in breast cancer. Oncoimmunology. (2017) 6:e1257452. 10.1080/2162402X.2016.1257452 28197375 PMC5283629

[150] Cancer Research UK. What is a Stem Cell Or Bone Marrow Transplant? (2019). Available online at: https://www.cancerresearchuk.org/about-cancer/cancer-in-general/treatment/bone-marrow-stem-cell-transplants/what-is (accessed May 24, 2021).

[151] Wolff M Shillington JM Rathbone C Piasecki SK Barnes B. Injections of concentrated bone marrow aspirate as treatment for discogenic pain: a retrospective analysis. BMC Musculoskelet Disord. (2020) 21:135. 10.1186/s12891-020-3126-7 32111220 PMC7049206

[152] Badalamenti G Fanale D Incorvaia L Barraco N Listì A Maragliano R. Role of tumor-infiltrating lymphocytes in patients with solid tumors: can a drop dig a stone? Cell Immunol. (2019) 343:103753. 10.1016/j.cellimm.2018.01.013 29395859

[153] Chen L Han X. Anti-PD-1/PD-L1 therapy of human cancer: past, present, and future. J Clin Invest. (2015) 125:3384–91. 10.1172/JCI80011 26325035 PMC4588282

[154] Lee M Li J Li J Fang S Zhang J Vo ATT. Tet2 inactivation enhances the antitumor activity of tumor-infiltrating lymphocytes. Cancer Res. (2021) 81:1965–76. 10.1158/0008-5472.CAN-20-3213 33589517 PMC8137576

[155] National Cancer Institute. Immune Checkpoint Inhibitors. (2019). Available online at: https://www.cancer.gov/about-cancer/treatment/types/immunotherapy/checkpoint-inhibitors (accessed May 24, 2021).

[156] Man YG Stojadinovic A Mason J Avital I Bilchik A Bruecher B. Tumor-infiltrating immune cells promoting tumor invasion and metastasis: existing theories. J Cancer. (2013) 4:84–95. 10.7150/jca.5482 23386907 PMC3564249

[157] Blazar BA Heppner GH. In situ lymphoid cells of mouse mammary tumors. J Immunol. (1978) 120:1876–80. 659881

[158] Zettergren JG Luberoff DE Pretlow TG. Separation of lymphocytes from disaggregated mouse malignant neoplasms by sedimentation in gradients of ficoll in tissue culture medium. J Immunol. (1973) 111:836–40. 4741299

[159] Spiess PJ Yang JC Rosenberg SA. In vivo antitumor activity of tumor-infiltrating lymphocytes expanded in recombinant interleukin-2. J Natl Cancer Inst. (1987) 79:1067–75. 3500355

[160] Rosenberg SA Spiess P Lafreniere R. A new approach to the adoptive immunotherapy of cancer with tumor-infiltrating lymphocytes. Science. (1986) 233:1318–21. 10.1126/science.3489291 3489291

[161] Geukes Foppen MH Donia M Svane IM Haanen JBAG. Tumor-infiltrating lymphocytes for the treatment of metastatic cancer. Mol Oncol. (2015) 9:1918–35. 10.1016/j.molonc.2015.10.018 26578452 PMC5528735

[162] Nirmal AJ Regan T Shih BB Hume DA Sims AH Freeman TC. Immune cell gene signatures for profiling the microenvironment of solid tumors. Cancer Immunol Res. (2018) 6:1388–400. 10.1158/2326-6066.CIR-18-0342 30266715

[163] Newman AM Liu CL Green MR Gentles AJ Feng W Xu Y. Robust enumeration of cell subsets from tissue expression profiles. Nat Methods. (2015) 12:453–7. 10.1038/nmeth.3337 25822800 PMC4739640

[164] Radvanyi LG. Tumor-infiltrating lymphocyte therapy: addressing prevailing questions. Cancer J. (2015) 21:450–64. 10.1097/PPO.0000000000000162 26588676

[165] Jiménez-Reinoso A Nehme-Álvarez D Domínguez-Alonso C Álvarez-Vallina L. Synthetic TILs: engineered tumor-infiltrating lymphocytes with improved therapeutic potential. Front Oncol. (2021) 10:593848. 10.3389/fonc.2020.593848 33680923 PMC7928359

[166] Schmidt-Wolf IGH Finke S Trojaneck B Denkena A Lefterova P Schwella N. Phase I clinical study applying autologous immunological effector cells transfected with the interleukin-1 gene in patients with metastatic renal cancer, colorectal cancer and lymphoma. Br J Cancer. (1999) 81:1009–16. 10.1038/sj.bjc.6690800 10576658 PMC2362953

[167] Semino C Martini L Queirolo P Cangemi G Costa R Alloisio A. Adoptive immunotherapy of advanced solid tumors: an eight year clinical experience. Anticancer Res. (1999) 19:5645–59. Available online at: https://europepmc.org/article/med/10697634 (accessed July 1, 2021). 10697634

[168] Young J Liu C Persechini P. Molecular mechanisms of lymphocyte-mediated killing. Braz J Med Biol Res. (1988) 21:1145–53. Available online at: https://pubmed.ncbi.nlm.nih.gov/3074838/ (accessed July 1, 2021). 3074838

[169] Hollister SJ. Porous scaffold design for tissue engineering. Nat Mater. (2005) 4:518–24. 10.1038/nmat1421 16003400

[170] Nichol JW Khademhosseini A. Modular tissue engineering: engineering biological tissues from the bottom up. Soft Matter. (2009) 5:1312–9. 10.1039/b814285h 20179781 PMC2826124

[171] US FDA. Complete List of Currently Approved Premarket Applications (PMAs). Road Canton, MA (2021). Available online at: https://www.fda.gov/media/76376/download (accessed May 26, 2021).

[172] Organogenesis Inc. Dermagraft^®^ (Human Fibroblast-Derived Dermal Substitute) Directions for Use. La Jolla, CA (2001). Available online at: https://dermagraft.com/pdf/Dermagraft-Directions-for-Use.pdf (accessed June 5, 2021).

[173] Yamato M Okano T. Cell sheet engineering. Mater Today. (2004) 7:42–7. 10.1016/S1369-7021(04)00234-2

[174] Matsuda N Shimizu T Yamato M Okano T. Tissue engineering based on cell sheet technology. Adv Mater. (2007) 19:3089–99. 10.1002/adma.20070197825855820

[175] Haraguchi Y Shimizu T Sasagawa T Sekine H Sakaguchi K Kikuchi T. Fabrication of functional three-dimensional tissues by stacking cell sheets in vitro. Nat Protoc. (2012) 7:850–8. 10.1038/nprot.2012.027 22481530

[176] Sekine H Shimizu T Sakaguchi K Dobashi I Wada M Yamato M. In vitro fabrication of functional three-dimensional tissues with perfusable blood vessels. Nat Commun. (2013) 4:1–10. 10.1038/ncomms2406 23360990 PMC3660653

[177] Sakaguchi K Shimizu T Horaguchi S Sekine H Yamato M Umezu M. In vitro engineering of vascularized tissue surrogates. Sci Rep. (2013) 3:1–7. 10.1038/srep01316 23419835 PMC3575583

[178] Kikuchi T Shimizu T Wada M Yamato M Okano T. Automatic fabrication of 3-dimensional tissues using cell sheet manipulator technique. Biomaterials. (2014) 35:2428–35. 10.1016/j.biomaterials.2013.12.014 24370007

[179] US FDA. Epicel (Cultured Epidermal Autografts) Directions for Use. Cambridge, MA (2016). Available online at: https://www.fda.gov/media/103138/download (accessed May 29, 2021).

[180] Zaulyanov L Kirsner RS. A review of a bi-layered living cell treatment (Apligraf ^®^) in the treatment of venous leg ulcers and diabetic foot ulcers. Clin Interv Aging. (2007) 2:93. 10.2147/ciia.2007.2.1.93 18044080 PMC2684073

[181] Chen M Przyborowski M Berthiaume F. Stem cells for skin tissue engineering and wound healing. Crit Rev Biomed Eng. (2009) 37:399–421. 10.1615/CritRevBiomedEng.v37.i4-5.50 20528733 PMC3223487

[182] Zuk PA Zhu M Mizuno H Huang J Futrell JW Katz AJ. Multilineage cells from human adipose tissue: Implications for cell-based therapies. Tissue Eng. (2001) 7:211–28. 10.1089/107632701300062859 11304456

[183] Traktuev DO Merfeld-Clauss S Li J Kolonin M Arap W Pasqualini R. Population of multipotent CD34-positive adipose stromal cells share pericyte and mesenchymal surface markers, reside in a periendothelial location, and stabilize endothelial networks. Circ Res. (2008) 102:77–85. 10.1161/CIRCRESAHA.107.159475 17967785

[184] Matsumoto D Sato K Gonda K Takaki Y Shigeura T Sato T. Cell-assisted lipotransfer: supportive use of human adipose-derived cells for soft tissue augmentation with lipoinjection. Tissue Eng. (2006) 12:3375–82. 10.1089/ten.2006.12.3375 17518674

[185] Glass GE Ferretti P. Adipose-derived stem cells in aesthetic surgery. Aesthetic Surg J. (2019) 39:423–38. 10.1093/asj/sjy160 29982396

[186] NIH. NCT04050111. The Evaluation Of Safety And Effectiveness Of Intraarticular Administration Of Autologous Stromal-Vascular Fraction Of Adipose Tissue Cells For Treatment Of Knee Joint Arthrosis. ClinicalTrialsGov (2019). Available online at: https://clinicaltrials.gov/ct2/show/NCT04050111?intr=stromal+vascular+fraction&draw=2&rank=2 (accessed June 6, 2021).

[187] NIH. NCT04238468. Scar Tissue Analysis After Intraoperative Application Of Stromal Vascular Fraction Cells Into Suture Line. ClinicalTrialsGov (2020). Available online at: https://clinicaltrials.gov/ct2/show/NCT04238468?intr=stromal+vascular+fraction&draw=2&rank=3 (accessed June 6, 2021).

[188] NIH. NCT04771442. Stem Cell Treatment Of Peyronie's Disease. ClinicalTrialsGov (2021). Available online at: https://clinicaltrials.gov/ct2/show/NCT04771442?intr=stromal+vascular+fraction&draw=2&rank=6 (accessed June 6, 2021).

[189] Ntege EH Sunami H Shimizu Y. Advances in regenerative therapy: a review of the literature and future directions. Regen Ther. (2020) 14:136–53. 10.1016/j.reth.2020.01.004 32110683 PMC7033303

[190] Bourin P Bunnell BA Casteilla L Dominici M Katz AJ March KL. Stromal cells from the adipose tissue-derived stromal vascular fraction and culture expanded adipose tissue-derived stromal/stem cells: a joint statement of the international federation for adipose therapeutics and science (IFATS) and the international society for cellular therapy (ISCT). Cytotherapy. (2013) 15:641–8. 10.1016/j.jcyt.2013.02.006 23570660 PMC3979435

[191] Guo J Nguyen A Banyard DA Fadavi D Toranto JD Wirth GA. Stromal vascular fraction: a regenerative reality? part 2: mechanisms of regenerative action. J Plast Reconstr Aesthet Surg. (2016) 69:180–8. 10.1016/j.bjps.2015.10.014 26546112

[192] Cutler C Antin JH. Peripheral blood stem cells for allogeneic transplantation: a review. Stem Cells. (2001) 19:108–17. 10.1634/stemcells.19-2-108 11239165

[193] ACS American Cancer Society. Stem Cell or Bone Marrow Transplant. (2020). Available online at: https://www.cancer.org/content/dam/CRC/PDF/Public/128.00.pdf (accessed September 18, 2021).

[194] Anderlini P Rizzo JD Nugent ML Schmitz N Champlin RE Horowitz MM. Peripheral blood stem cell donation: an analysis from the international bone marrow transplant registry (IBMTR) and European group for blood and marrow transplant (EBMT) databases. Bone Marrow Transplant. (2001) 27:689–92. 10.1038/sj.bmt.1702875 11360107

[195] Rindy LJ Chambers AR. Bone Marrow Aspiration Biopsy. Treasure Island, FL: StatPearls Publishing (2021). Available online at: http://www.ncbi.nlm.nih.gov/pubmed/32644658 (accessed May 30, 2021).

[196] Gorin NC. Bone marrow harvesting for HSCT. In: The EBMT Handbook: Hematopoietic Stem Cell Transplantation and Cellular Therapies. Cham, Switzerland: Springer International Publishing. p. 109–15. 10.1007/978-3-030-02278-5_14

[197] Pang WW Price EA Sahoo D Beerman I Maloney WJ Rossi DJ. Human bone marrow hematopoietic stem cells are increased in frequency and myeloid-biased with age. Proc Natl Acad Sci U S A. (2011) 108:20012–7. 10.1073/pnas.1116110108 22123971 PMC3250139

[198] Imam MA Mahmoud SSS Holton J Abouelmaati D Elsherbini Y Snow M. systematic review of the concept and clinical applications of bone marrow aspirate concentrate in orthopaedics. Sicot J. (2017) 3:17. 10.1051/sicotj/2017007 29792397 PMC5966837

[199] Amouzegar A Dey BR Spitzer TR. peripheral blood or bone marrow stem Cells? practical considerations in hematopoietic stem cell transplantation. Transfus Med Rev. (2019) 33:43–50. 10.1016/j.tmrv.2018.11.003 30528986

[200] Ho AD Haas R Champlin RE. Hematopoietic Stem Cell Transplantation. Treasure Island, FL: CRC Press (2000).

[201] Teipel R Oelschlägel U Wetzko K Schmiedgen M Kramer M Rücker-Braun E. Differences in cellular composition of peripheral blood stem cell grafts from healthy stem cell donors mobilized with either granulocyte colony-stimulating factor (G-CSF) alone or G-CSF and plerixafor. Biol Blood Marrow Transplant. (2018) 24:2171–7. 10.1016/j.bbmt.2018.06.023 29935214

[202] Gyger M Stuart RK Perreault C. Immunobiology of allogeneic peripheral blood mononuclear cells mobilized with granulocyte-colony stimulating factor. Bone Marrow Transplant. (2000) 26:1–16. 10.1038/sj.bmt.1702464 10918400

[203] Körbling M Anderlini P. Peripheral blood stem cell versus bone marrow allotransplantation: does the source of hematopoietic stem cells matter? Blood. (2001) 98:2900–8. 10.1182/blood.V98.10.2900 11698269

[204] Yasui K Matsumoto K Hirayama F Tani Y Nakano T. Differences between peripheral blood and cord blood in the kinetics of lineage-restricted hematopoietic cells: implications for delayed platelet recovery following cord blood transplantation. Stem Cells. (2003) 21:143–51. 10.1634/stemcells.21-2-143 12634410

[205] Robin M Ruggeri A Labopin M Niederwieser D Tabrizi R Sanz G. Comparison of unrelated cord blood and peripheral blood stem cell transplantation in adults with myelodysplastic syndrome after reduced-intensity conditioning regimen: a collaborative study from eurocord (Cord blood committee of cellular therapy & immunobiology working party of EBMT) and chronic malignancies working party. Biol Blood Marrow Transplant. (2015) 21:489–95. 10.1016/j.bbmt.2014.11.675 25529382

[206] Passweg JR Baldomero H Gratwohl A Bregni M Cesaro S Dreger P. The EBMT activity survey: 1990-2010. Bone Marrow Transplant. (2012) 47:906–23. 10.1038/bmt.2012.66 22543746

[207] Passweg JR Baldomero H Bader P Bonini C Cesaro S Dreger P. Hematopoietic stem cell transplantation in Europe 2014: more than 40 000 transplants annually. Bone Marrow Transplant. (2016) 51:786–92. 10.1038/bmt.2016.20 26901709 PMC4895175

[208] Lee DH Ryu KJ Kim JW Kang KC Choi YR. Bone marrow aspirate concentrate and platelet-rich plasma enhanced bone healing in distraction osteogenesis of the Tibia. Clin Orthop Relat Res. (2014) 472:3789–97. 10.1007/s11999-014-3548-3 24599650 PMC4397746

[209] El Kadiry AEH Lumbao C Rafei M Shammaa R. Autologous BMAC therapy improves spinal degenerative joint disease in lower back pain patients. Front Med. (2021) 8:622573. 10.3389/fmed.2021.622573 33816523 PMC8012529

[210] Kouroupis D Ahari AF Correa D Shammaa R. Intralesional injection of bone marrow aspirate concentrate for the treatment of osteonecrosis of the knee secondary to systemic lupus erythematosus: a case report. Front Bioeng Biotechnol. (2020) 8:202. 10.3389/fbioe.2020.00202 32266233 PMC7100546

[211] Sugaya H Yoshioka T Kato T Taniguchi Y Kumagai H Hyodo K. Comparative analysis of cellular and growth factor composition in bone marrow aspirate concentrate and platelet-rich plasma. Bone Marrow Res. (2018) 2018:1–9. 10.1155/2018/1549826 29682351 PMC5845506

[212] Holton J Imam M Ward J Snow M. The basic science of bone marrow aspirate concentrate in chondral injuries. Orthop Rev. (2016) 8:80–4. 10.4081/or.2016.6659 27761221 PMC5066111

[213] Dragoo JL Guzman RA. Evaluation of the consistency and composition of commercially available bone marrow aspirate concentrate systems. Orthop J Sport Med. (2020) 8:1–8. 10.1177/2325967119893634 32010732 PMC6970477

[214] Riley RS Hogan TF Pavot DR Forysthe R Massey D Smith E. Pathologist's perspective on bone marrow aspiration and biopsy: I. performing a bone marrow examination. J Clin Lab Anal. (2004) 18:70–90. 10.1002/jcla.20008 15065211 PMC6807972

[215] Chahla J Mannava S Cinque ME Geeslin AG Codina D LaPrade RF. Bone marrow aspirate concentrate harvesting and processing technique. Arthrosc Tech. (2017) 6:e441–5. 10.1016/j.eats.2016.10.024 28580265 PMC5443590

[216] Kim GB Seo MS Park WT Lee GW. Bone marrow aspirate concentrate: its uses in osteoarthritis. Int J Mol Sci. (2020) 21:3224. 10.3390/ijms21093224 32370163 PMC7247342

[217] Pavlovic V Ciric M Jovanovic V Stojanovic P. Platelet rich plasma: a short overview of certain bioactive components. Open Med. (2016) 11:242–7. 10.1515/med-2016-0048 28352802 PMC5329835

[218] Dhurat R Sukesh M. Principles and methods of preparation of platelet-rich plasma: a review and author′s perspective. J Cutan Aesthet Surg. (2014) 7:189. 10.4103/0974-2077.150734 25722595 PMC4338460

[219] Kojok K El-Kadiry AE-H Merhi Y. Role of NF-κB in platelet function. Int J Mol Sci. (2019) 20:4185. 10.3390/ijms20174185 31461836 PMC6747346

[220] El-Kadiry AE-H Merhi Y. The role of the proteasome in platelet function. Int J Mol Sci. (2021) 22:3999. 10.3390/ijms22083999 33924425 PMC8069084

[221] Fitzpatrick J Bulsara MK McCrory PR Richardson MD Zheng MH. Analysis of platelet-rich plasma extraction: variations in platelet and blood components between 4 common commercial kits. Orthop J Sport Med. (2017) 5:1–8. 10.1177/2325967116675272 28210651 PMC5302100

[222] Kitamura Y Suzuki M Tsukioka T Isobe K Tsujino T Watanabe T. Spectrophotometric determination of platelet counts in platelet-rich plasma. Int J Implant Dent. (2018) 4:1–8. 10.1186/s40729-018-0140-8 30276491 PMC6167270

[223] De Oliveira LA Soares RO Buzzi M Mourao CFAB Kawase T Kuckelhaus SAS. Cell and platelet composition assays by flow cytometry: basis for new platelet-rich fibrin methodologies. J Biol Regul Homeost Agents. (2020) 34:1379–90. 10.23812/20-278-A 32867466

[224] Everts P Onishi K Jayaram P Lana JF Mautner K. Platelet-rich plasma: new performance understandings and therapeutic considerations in 2020. Int J Mol Sci. (2020) 21:1–36. 10.20944/preprints202010.0069.v1 33096812 PMC7589810

[225] Alsousou J Ali A Willett K Harrison P. The role of platelet-rich plasma in tissue regeneration. Platelets. (2013) 24:173–82. 10.3109/09537104.2012.684730 22647081

[226] Redler LH Thompson SA Hsu SH Ahmad CS Levine WN. Platelet-rich plasma therapy: a systematic literature review and evidence for clinical use. Phys Sportsmed. (2011) 39:42–51. 10.3810/psm.2011.02.1861 21378486

[227] US FDA. Regulatory Considerations for Human Cells, Tissues, and Cellular and Tissue-Based Products: Minimal Manipulation Homologous Use - Guidance for Industry and Food and Drug Administration Staff. (2020). Available online at: https://www.fda.gov/media/109176/download (accessed June 20, 2021).

[228] US FDA. Same Surgical Procedure Exception under 21 CFR 1271.15(b): Questions Answers Regarding the Scope of the Exception; Guidance for Industry. (2017). Available online at: https://www.fda.gov/media/89920/download (accessed June 20, 2021).

[229] Bloomberg Law. INSIGHT: FDA's Aggressive Enforcement Against Stem Cell Companies Starting to Ripen. US Law Week. (2019). Available online at: https://news.bloomberglaw.com/us-law-week/insight-fdas-aggressive-enforcement-against-stem-cell-companies-starting-to-ripen (accessed July 24, 2021).

[230] US FDA. Statement on stem cell clinic permanent injunction FDA's ongoing efforts to protect patients from risks of unapproved products. FDA Statement. (2019). Available online at: https://www.fda.gov/news-events/press-announcements/statement-stem-cell-clinic-permanent-injunction-and-fdas-ongoing-efforts-protect-patients-risks (accessed July 24, 2021).

[231] Los Angeles Times. Column: Judge Hands FDA a Loss On Stem Cell Clinics. (2020). Available online at: https://www.latimes.com/business/story/2020-01-28/judge-fda-stem-cell-clinics (accessed July 25, 2021).

[232] United States District Court. United States of America v. California Stem Cell Treatment Center, Inc., et al. California (2020). Available online at: https://s3.documentcloud.org/documents/6682558/Stem-Cell-Berman-Ruling.pdf

[233] US FDA. Apligraf^®^ (Graftskin) Prescribing Information. Canton, MA (2001). Available online at: https://www.accessdata.fda.gov/cdrh_docs/pdf/P950032S016c.pdf (accessed June 5, 2021).

[234] Mao AS Mooney DJ. Regenerative medicine: current therapies and future directions. Proc Natl Acad Sci U S A. (2015) 112:14452–9. 10.1073/pnas.1508520112 26598661 PMC4664309

[235] DiMasi JA Hansen RW Grabowski HG. The price of innovation: new estimates of drug development costs. J Health Econ. (2003) 22:151–85. 10.1016/S0167-6296(02)00126-1 12606142

[236] Avorn J. The $2.6 billion pill — methodologic and policy considerations. N Engl J Med. (2015) 372:1877–9. 10.1056/NEJMp1500848 25970049

[237] Beall RF Hwang TJ Kesselheim AS. Pre-market development times for biologic versus small-molecule drugs. Nat Biotechnol. (2019) 37:708–11. 10.1038/s41587-019-0175-2 31213674

[238] Jarrige M Frank E Herardot E Martineau S Darle A Benabides M. The future of regenerative medicine: cell therapy using pluripotent stem cells and acellular therapies based on extracellular vesicles. Cells. (2021) 10:240. 10.3390/cells10020240 33513719 PMC7912181

[239] Jayabalan P Hagerty S Cortazzo MH. The use of platelet-rich plasma for the treatment of osteoarthritis. Phys Sportsmed. (2014) 42:53–62. 10.3810/psm.2014.09.2076 25295767

[240] Miller LE Parrish WR Roides B Bhattacharyya S. Efficacy of platelet-rich plasma injections for symptomatic tendinopathy: Systematic review and meta-analysis of randomised injection-controlled trials. BMJ Open Sport Exerc Med. (2017) 3:e000237. 10.1136/bmjsem-2017-000237 29177072 PMC5687544

[241] Le ADK Enweze L DeBaun MR Dragoo JL. Current clinical recommendations for use of platelet-rich plasma. Curr Rev Musculoskelet Med. (2018) 11:624–34. 10.1007/s12178-018-9527-7 30353479 PMC6220007

[242] Vavken P Sadoghi P Palmer M Rosso C Mueller AM Szoelloesy G. Platelet-rich plasma reduces retear rates after arthroscopic repair of small- and medium-sized rotator cuff tears but is not cost-effective. Am J Sports Med. (2015) 43:3071–6. 10.1177/0363546515572777 25767267

[243] Dallari D Stagni C Rani N Sabbioni G Pelotti P Torricelli P. Ultrasound-guided injection of platelet-rich plasma and hyaluronic acid, separately and in combination, for hip osteoarthritis. Am J Sports Med. (2016) 44:664–71. 10.1177/0363546515620383 26797697

[244] Cole BJ Karas V Hussey K Pilz K Fortier LA. Hyaluronic acid versus platelet-rich plasma. Am J Sports Med. (2017) 45:339–46. 10.1177/0363546516665809 28146403

[245] A Hamid MS Mohamed Ali MR Yusof A George J Lee LPC. Platelet-rich plasma injections for the treatment of hamstring injuries: a randomized controlled trial. Am J Sports Med. (2014) 42:2410–8. 10.1177/0363546514541540 25073598

[246] Sundman EA Cole BJ Fortier LA. Growth factor and catabolic cytokine concentrations are influenced by the cellular composition of platelet-rich plasma. Am J Sports Med. (2011) 39:2135–40. 10.1177/0363546511417792 21846925

[247] Azuma K Yamanaka S. Recent policies that support clinical application of induced pluripotent stem cell-based regenerative therapies. Regen Ther. (2016) 4:36–47. 10.1016/j.reth.2016.01.009 31245486 PMC6581825

[248] Andia I Maffulli N Burgos-Alonso N. Stromal vascular fraction technologies and clinical applications. Expert Opin Biol Ther. (2019) 19:1289–305. 10.1080/14712598.2019.1671970 31544555

[249] Rigotti G Marchi A Galiè M Baroni G Benati D Krampera M. Clinical treatment of radiotherapy tissue damage by lipoaspirate transplant: a healing process mediated by adipose-derived adult stem cells. Plast Reconstr Surg. (2007) 119:1409–22. 10.1097/01.prs.0000256047.47909.71 17415234

[250] Hudetz D Borić I Rod E Jeleč Ž Radić A Vrdoljak T. The effect of intra-articular injection of autologous microfragmented fat tissue on proteoglycan synthesis in patients with knee osteoarthritis. Genes. (2017) 8:270. 10.3390/genes8100270 29027984 PMC5664120

[251] Yokota N Yamakawa M Shirata T Kimura T Kaneshima H. Clinical results following intra-articular injection of adipose-derived stromal vascular fraction cells in patients with osteoarthritis of the knee. Regen Ther. (2017) 6:108–12. 10.1016/j.reth.2017.04.002 30271845 PMC6134915

[252] Moon KC Chung HY Han SK Jeong SH Dhong ES. Possibility of injecting adipose-derived stromal vascular fraction cells to accelerate microcirculation in ischemic diabetic feet: a pilot study. Int J Stem Cells. (2019) 12:107–13. 10.15283/ijsc18101 30836733 PMC6457712

[253] Giori A Tremolada C Vailati R Navone S. Marfia G Caplan A. Recovery of function in anal incontinence after micro-fragmented fat graft (Lipogems^®^) injection: two years follow up of the first 5 cases. CellR4. (2015) 3:e1544. Available online at: http://www.cellr4.org/wp-content/uploads/sites/2/2015/03/CellR4-2015-3-2-e1544-Alberto-Giori.pdf (accessed June 12, 2021).

[254] Sautereau N Daumas A Truillet R Jouve E Magalon J Veran J. Efficacy of autologous microfat graft on facial handicap in systemic sclerosis patients. Plast Reconstr Surg-Glob Open. (2016) 4:e660. 10.1097/GOX.0000000000000621 27257590 PMC4874304

[255] Frese L Dijkman PE Hoerstrup SP. Adipose tissue-derived stem cells in regenerative medicine. Transfus Med Hemotherapy. (2016) 43:268–74. 10.1159/000448180 27721702 PMC5040903

[256] NIH. Recruiting, Active, Not Recruiting Studies ADSC. ClinicalTrialsGov (2021). Available online at: https://clinicaltrials.gov/ct2/results?intr=ADSC&Search=Apply&recrs=a&recrs=d&age_v=&gndr=&type=&rslt= (accessed June 13, 2021).

[257] Houtgraaf JH Den Dekker WK Van Dalen BM Springeling T De Jong R Van Geuns RJ. First experience in humans using adipose tissue-derived regenerative cells in the treatment of patients with ST-segment elevation myocardial infarction. J Am Coll Cardiol. (2012) 59:539–40. 10.1016/j.jacc.2011.09.065 22281257

[258] Fodor PB Paulseth SG. Adipose derived stromal cell (ADSC) injections for pain management of osteoarthritis in the human knee joint. Aesthetic Surg J. (2016) 36:229–36. 10.1093/asj/sjv135 26238455

[259] Spasovski D Spasovski V Baščarević Z Stojiljković M Vreća M Andelković M. Intra-articular injection of autologous adipose-derived mesenchymal stem cells in the treatment of knee osteoarthritis. J Gene Med. (2018) 20:1–8. 10.1002/jgm.3002 29243283

[260] Carstens MH Gómez A Cortés R Turner E Pérez C Ocon M. Non-reconstructable peripheral vascular disease of the lower extremity in ten patients treated with adipose-derived stromal vascular fraction cells. Stem Cell Res. (2017) 18:14–21. 10.1016/j.scr.2016.12.001 27984756

[261] Papa DN Di Luca G Sambataro D Zaccara E Maglione W Gabrielli A. Regional implantation of autologous adipose tissue-derived cells induces a prompt healing of long-lasting indolent digital ulcers in patients with systemic sclerosis. Cell Transplant. (2015) 24:2297–305. 10.3727/096368914X685636 25506730

[262] Granel B Daumas A Jouve E Harlé JR Nguyen PS Chabannon C. Safety, tolerability and potential efficacy of injection of autologous adipose-derived stromal vascular fraction in the fingers of patients with systemic sclerosis: an open-label phase I trial. Ann Rheum Dis. (2015) 74:2175–82. 10.1136/annrheumdis-2014-205681 25114060 PMC4680117

[263] Andjelkov K Sforza M Barisic G Soldatovic I Hiranyakas A Krivokapic Z. Novel method for treatment of chronic anal fissure: adipose-derived regenerative cells – a pilot study. Color Dis. (2017) 19:570–5. 10.1111/codi.13555 28574663

[264] Choi JY Kim TH Yang JD Suh JS Kwon TG. Adipose-derived regenerative cell injection therapy for postprostatectomy incontinence: a phase i clinical study. Yonsei Med J. (2016) 57:1152–8. 10.3349/ymj.2016.57.5.1152 27401646 PMC4960381

[265] Li ZJ Yang E Li YZ Liang ZY Huang JZ Yu NZ. Application and prospect of adipose stem cell transplantation in treating lymphedema. World J Stem Cells. (2020) 12:676–87. 10.4252/wjsc.v12.i7.676 32843921 PMC7415250

[266] Tappenbeck N Schröder HM Niebergall-Roth E Hassinger F Dehio U Dieter K. In vivo safety profile and biodistribution of GMP-manufactured human skin-derived ABCB5-positive mesenchymal stromal cells for use in clinical trials. Cytotherapy. (2019) 21:546–60. 10.1016/j.jcyt.2018.12.005 30878384 PMC6513723

[267] Yamada Y Ueda M Hibi H Baba S. A novel approach to periodontal tissue regeneration with mesenchymal stem cells and platelet-rich plasma using tissue engineering technology: A clinical case report. Int J Periodontics Restorative Dent. (2006) 26:363–9. Available online at: http://www.ncbi.nlm.nih.gov/pubmed/16939018 (accessed September 13, 2019). 16939018

[268] Kyriakidis T Iosifidis M Michalopoulos E Melas I Stavropoulos-Giokas C Verdonk R. Good mid-term outcomes after adipose-derived culture-expanded mesenchymal stem cells implantation in knee focal cartilage defects. Knee Surgery, Sport Traumatol Arthrosc. (2020) 28:502–8. 10.1007/s00167-019-05688-9 31493012

[269] Redondo LM García V Peral B Verrier A Becerra J Sánchez A. Repair of maxillary cystic bone defects with mesenchymal stem cells seeded on a cross-linked serum scaffold. J Cranio-Maxillofacial Surg. (2018) 46:222–9. 10.1016/j.jcms.2017.11.004 29229365

[270] Lu D Chen B Liang Z Deng W Jiang Y Li S. Comparison of bone marrow mesenchymal stem cells with bone marrow-derived mononuclear cells for treatment of diabetic critical limb ischemia and foot ulcer: a double-blind, randomized, controlled trial. Diabetes Res Clin Pract. (2011) 92:26–36. 10.1016/j.diabres.2010.12.010 21216483

[271] Hahn J-Y Cho H-J Kang H-J Kim T-S Kim M-H Chung J-H. Pre-treatment of mesenchymal stem cells with a combination of growth factors enhances gap junction formation, cytoprotective effect on cardiomyocytes, and therapeutic efficacy for myocardial infarction. J Am Coll Cardiol. (2008) 51:933–43. 10.1016/j.jacc.2007.11.040 18308163

[272] Haynesworth SE Baber MA Caplan AI. Cytokine expression by human marrow-derived mesenchymal progenitor cells in vitro: Effects of dexamethasone and IL-1α. J Cell Physiol. (1996) 166:585–92. 10.1002/(SICI)1097-4652(199603)166:3<585::AID-JCP13>3.0.CO;2-6 8600162

[273] Min J-Y Sullivan MF Yang Y Zhang J-P Converso KL Morgan JP. Significant improvement of heart function by cotransplantation of human mesenchymal stem cells and fetal cardiomyocytes in postinfarcted pigs. Ann Thorac Surg. (2002) 74:1568–75. 10.1016/S0003-4975(02)03952-8 12440610

[274] Centeno C Pitts J Al-Sayegh H Freeman M. Efficacy of autologous bone marrow concentrate for knee osteoarthritis with and without adipose graft. Biomed Res Int. (2014) 2014:1–9. 10.1155/2014/370621 25276781 PMC4170694

[275] Shapiro SA Kazmerchak SE Heckman MG Zubair AC O'Connor MI A. Prospective, single-blind, placebo-controlled trial of bone marrow aspirate concentrate for knee osteoarthritis. Am J Sports Med. (2017) 45:82–90. 10.1177/0363546516662455 27566242

[276] NIH. Search of: Recruiting, Active, not recruiting Studies BMAC. ClinicalTrialsGov (2021). Available online at: https://clinicaltrials.gov/ct2/results?intr=BMAC&Search=Apply&recrs=a&recrs=d&age_v=&gndr=&type=&rslt= (accessed June 20, 2021).

[277] Weel VV van Tongeren RB van Hinsbergh VWM van Bockel JH Quax PHA. Vascular growth in ischemic limbs: a review of mechanisms and possible therapeutic stimulation. Ann Vasc Surg. (2008) 22:582–97. 10.1016/j.avsg.2008.02.017 18504100

[278] Shireman PK. The chemokine system in arteriogenesis and hind limb ischemia. J Vasc Surg. (2007) 45:A48-56. 10.1016/j.jvs.2007.02.030 PMC2680944 17544024

[279] US National Center for Biotechnology Information. Diseases of the Immune System. Genes Dis. (1998). Available online at: https://www.ncbi.nlm.nih.gov/books/NBK22243/ (accessed June 20, 2021).

[280] Richard-Eaglin A Smallheer BA. Immunosuppressive/autoimmune disorders. Nurs Clin North Am. (2018) 53:319–34. 10.1016/j.cnur.2018.04.002 30099999

[281] Dazzi F Van Laar JM Cope A Tyndall A. Cell therapy for autoimmune diseases. Arthritis Res Ther. (2007) 9:206. 10.1186/ar2128 17367542 PMC1906794

[282] Fang B Song Y Liao L Zhang Y Zhao RC. Favorable response to human adipose tissue-derived mesenchymal stem cells in steroid-refractory acute graft-versus-host disease. Transplant Proc. (2007) 39:3358–62. 10.1016/j.transproceed.2007.08.103 18089385

[283] Lee WY Park KJ Cho YB Yoon SN Song KH Kim DS. Autologous adipose tissue-derived stem cells treatment demonstrated favorable and sustainable therapeutic effect for crohn's fistula. Stem Cells. (2013) 31:2575–81. 10.1002/stem.1357 23404825

[284] Comella K Parlo M Daly R Dominessy K. First-in-man intravenous implantation of stromal vascular fraction in psoriasis: a case study. Int Med Case Rep J. (2018) 11:59–64. 10.2147/IMCRJ.S163612 29606893 PMC5868735

[285] Onesti MG Fioramonti P Carella S Fino P Marchese C Scuderi N. Improvement of mouth functional disability in systemic sclerosis patients over one year in a trial of fat transplantation versus adipose-derived stromal cells. Stem Cells Int. (2016) 2016:2416192. 10.1155/2016/2416192 26880939 PMC4736419

[286] Badsha H Harifi G Murrell WD. Platelet rich plasma for treatment of rheumatoid arthritis: case series and review of literature. Case Rep Rheumatol. (2020) 2020:1–7. 10.1155/2020/8761485 32082684 PMC7021456

[287] Brunstein CG Miller JS Cao Q McKenna DH Hippen KL Curtsinger J. Infusion of ex vivo expanded T regulatory cells in adults transplanted with umbilical cord blood: safety profile and detection kinetics. Blood. (2011) 117:1061–70. 10.1182/blood-2010-07-293795 20952687 PMC3035067

[288] Domogalla MP Rostan PV Raker VK Steinbrink K. Tolerance through education: how tolerogenic dendritic cells shape immunity. Front Immunol. (2017) 8:1764. 10.3389/fimmu.2017.01764 29375543 PMC5770648

[289] Bell GM Anderson AE Diboll J Reece R Eltherington O Harry RA. Autologous tolerogenic dendritic cells for rheumatoid and inflammatory arthritis. Ann Rheum Dis. (2017) 76:227–34. 10.1136/annrheumdis-2015-208456 27117700 PMC5264217

[290] Phillips BE Garciafigueroa Y Trucco M Giannoukakis N. Clinical tolerogenic dendritic cells: exploring therapeutic impact on human autoimmune disease. Front Immunol. (2017) 8:1279. 10.3389/fimmu.2017.01279 29075262 PMC5643419

[291] Choudhary P Rickels MR Senior PA Vantyghem M-C Maffi P Kay TW. Evidence-informed clinical practice recommendations for treatment of type 1 diabetes complicated by problematic hypoglycemia. Diabetes Care. (2015) 38:1016–29. 10.2337/dc15-0090 25998294 PMC4439532

[292] Jhaveri KS Schlam I Holtzman NG Peravali M Richardson PK Dahiya S. Safety and efficacy of CAR T cells in a patient with lymphoma and a coexisting autoimmune neuropathy. Blood Adv. (2020) 4:6019–22. 10.1182/bloodadvances.2020003176 33284942 PMC7724903

[293] Maldini CR Ellis GI Riley JL CAR T. Cells for infection, autoimmunity and allotransplantation. Nat Rev Immunol. (2018) 18:605–16. 10.1038/s41577-018-0042-2 30046149 PMC6505691

[294] Schirrmacher V. From chemotherapy to biological therapy: a review of novel concepts to reduce the side effects of systemic cancer treatment. Int J Oncol. (2019) 54:407–19. 10.3892/ijo.2018.4661 30570109 PMC6317661

[295] Yu B Jiang T Liu D. BCMA-targeted immunotherapy for multiple myeloma. J Hematol Oncol. (2020) 13:1–24. 10.1186/s13045-020-00962-7 32943087 PMC7499842

[296] Timmerman JM Czerwinski DK Davis TA Hsu FJ Benike C Hao ZM. Idiotype-pulsed dendritic cell vaccination for B-cell lymphoma: clinical and immune responses in 35 patients. Blood. (2002) 99:1517–26. 10.1182/blood.V99.5.1517 11861263

[297] Rosenblatt J Avivi I Vasir B Uhl L Munshi NC Katz T. Vaccination with dendritic cell/tumor fusions following autologous stem cell transplant induces immunologic and clinical responses in multiple myeloma patients. Clin Cancer Res. (2013) 19:3640–8. 10.1158/1078-0432.CCR-13-0282 23685836 PMC3755905

[298] Anguille S Van De Velde AL Smits EL Van Tendeloo VF Juliusson G Cools N. Dendritic cell vaccination as postremission treatment to prevent or delay relapse in acute myeloid leukemia. Blood. (2017) 130:1713–21. 10.1182/blood-2017-04-780155 28830889 PMC5649080

[299] Polyzoidis S Tuazon J Brazil L Beaney R Al-Sarraj ST Doey L. Active dendritic cell immunotherapy for glioblastoma: current status and challenges. Br J Neurosurg. (2015) 29:197–205. 10.3109/02688697.2014.994473 25541743

[300] Dillman RO Cornforth AN Depriest C McClay EF Amatruda TT De Leon C. Tumor stem cell antigens as consolidative active specific immunotherapy: A randomized phase II trial of dendritic cells versus tumor cells in patients with metastatic melanoma. J Immunother. (2012) 35:641–9. 10.1097/CJI.0b013e31826f79c8 22996370

[301] Schreibelt G Bol KF Westdorp H Wimmers F Aarntzen EHJG Duiveman-De Boer T. Effective clinical responses in metastatic melanoma patients after vaccination with primary myeloid dendritic cells. Clin Cancer Res. (2016) 22:2155–66. 10.1158/1078-0432.CCR-15-2205 26712687

[302] Tel J Erik HJGA Baba T Schreibelt G Schulte BM Benitez-Ribas D. Natural human plasmacytoid dendritic cells induce antigen-specific T-cell responses in melanoma patients. Cancer Res. (2013) 73:1063–75. 10.1158/0008-5472.CAN-12-2583 23345163

[303] Akasaki Y Kikuchi T Homma S Koido S Ohkusa T Tasaki T. Phase I/II trial of combination of temozolomide chemotherapy and immunotherapy with fusions of dendritic and glioma cells in patients with glioblastoma. Cancer Immunol Immunother. (2016) 65:1499–509. 10.1007/s00262-016-1905-7 27688162 PMC11028634

[304] Rosenblatt J Stone RM Uhl L Neuberg D Joyce R Levine JD. Individualized vaccination of AML patients in remission is associated with induction of antileukemia immunity and prolonged remissions. Sci Transl Med. (2016) 8:368ra171. 10.1126/scitranslmed.aag1298 27928025 PMC5800949

[305] Raje N Berdeja J Lin Y Siegel D Jagannath S Madduri D. Anti-BCMA CAR T-Cell Therapy bb2121 in relapsed or refractory multiple myeloma. N Engl J Med. (2019) 380:1726–37. 10.1056/NEJMoa1817226 31042825 PMC8202968

[306] Rodríguez Pérez Campillo-Davo D Van Tendeloo VFI Benítez-Ribas D. Cellular immunotherapy: a clinical state-of-the-art of a new paradigm for cancer treatment. Clin Transl Oncol. (2020) 22:1923–37. 10.1007/s12094-020-02344-4 32266674

[307] Zhang H Gao L Liu L Wang J Wang S Gao L. A Bcma and CD19 Bispecific CAR-T for relapsed and refractory multiple myeloma. Blood. (2019) 134:3147. 10.1182/blood-2019-13105616462024

[308] Morgan RA Dudley ME Wunderlich JR Hughes MS Yang JC Sherry RM. Cancer regression in patients after transfer of genetically engineered lymphocytes. Science. (2006) 314:126–9. 10.1126/science.1129003 16946036 PMC2267026

[309] Johnson LA Morgan RA Dudley ME Cassard L Yang JC Hughes MS. Gene therapy with human and mouse T-cell receptors mediates cancer regression and targets normal tissues expressing cognate antigen. Blood. (2009) 114:535–46. 10.1182/blood-2009-03-211714 19451549 PMC2929689

[310] Robbins PF Kassim SH Tran TLN Crystal JS Morgan RA Feldman SA. A pilot trial using lymphocytes genetically engineered with an NY-ESO-1-reactive T-cell receptor: long-term follow-up and correlates with response. Clin Cancer Res. (2015) 21:1019–27. 10.1158/1078-0432.CCR-14-2708 25538264 PMC4361810

[311] Mackall C Tap WD Glod J Druta M Chow WA Araujo DM. Open label, non-randomized, multi-cohort pilot study of genetically engineered NY-ESO-1 specific NY-ESO-1c259t in HLA-A2+ patients with synovial sarcoma (NCT01343043). J Clin Oncol. (2017) 35:3000. 10.1200/JCO.2017.35.15_suppl.3000

[312] Rosenberg SA Packard BS Aebersold PM Solomon D Topalian SL Toy ST. Use of tumor-infiltrating lymphocytes and interleukin-2 in the immunotherapy of patients with metastatic melanoma. N Engl J Med. (1988) 319:1676–80. 10.1056/NEJM198812223192527 3264384

[313] Dudley ME Wunderlich JR Yang JC Sherry RM Topalian SL Restifo NP. Adoptive cell transfer therapy following non-myeloablative but lymphodepleting chemotherapy for the treatment of patients with refractory metastatic melanoma. J Clin Oncol. (2005) 23:2346–57. 10.1200/JCO.2005.00.240 15800326 PMC1475951

[314] Rosenberg SA Lotze MT Muul LM Leitman S Chang AE Ettinghausen SE. Observations on the systemic administration of autologous lymphokine-activated killer cells and recombinant interleukin-2 to patients with metastatic cancer. N Engl J Med. (1985) 313:1485–92. 10.1056/NEJM198512053132327 3903508

[315] Rosenberg SA Lotze MT Yang JC Topalian SL Chang AE Schwartzentruber DJ. Prospective randomized trial of high-dose interleukin-2 alone or in conjunction with lymphokine-activated killer cells for the treatment of patients with advanced cancer. J Natl Cancer Inst. (1993) 85:622–32. 10.1093/jnci/85.8.622 8468720

[316] Dillman RO Duma CM Schiltz PM DePriest C Ellis RA Okamoto K. Intracavitary placement of autologous lymphokine-activated killer (LAK) cells after resection of recurrent glioblastoma. J Immunother. (2004) 27:398–404. 10.1097/00002371-200409000-00009 15314549

[317] Robbins PF Dudley ME Wunderlich J El-Gamil M Li YF Zhou J. Cutting edge: persistence of transferred lymphocyte clonotypes correlates with cancer regression in patients receiving cell transfer therapy. J Immunol. (2004) 173:7125–30. 10.4049/jimmunol.173.12.7125 15585832 PMC2175171

[318] Weng DS Zhou J Zhou QM Zhao M Wang QJ Huang LX. Minimally invasive treatment combined with cytokine-induced killer cells therapy lower the short-term recurrence rates of hepatocellular carcinomas. J Immunother. (2008) 31:63–71. 10.1097/CJI.0b013e31815a121b 18157013

[319] Hui D Qiang L Jian W Ti Z Da-Lu K. A randomized, controlled trial of postoperative adjuvant cytokine-induced killer cells immunotherapy after radical resection of hepatocellular carcinoma. Dig Liver Dis. (2009) 41:36–41. 10.1016/j.dld.2008.04.007 18818130

[320] Lee JH Lee JH Lim YS Yeon JE Song TJ Yu SJ. Adjuvant immunotherapy with autologous cytokine-induced killer cells for hepatocellular carcinoma. Gastroenterology. (2015) 148:1383–91.e6. 10.1053/j.gastro.2015.02.055 25747273

[321] Sangiolo D Martinuzzi E Todorovic M Vitaggio K Vallario A Jordaney N. Alloreactivity and anti-tumor activity segregate within two distinct subsets of cytokine-induced killer (CIK) cells: implications for their infusion across major HLA barriers. Int Immunol. (2008) 20:841–8. 10.1093/intimm/dxn042 18469328

[322] Curti A Ruggeri L D'Addio A Bontadini A Dan E Motta MR. Successful transfer of alloreactive haploidentical KIR ligand-mismatched natural killer cells after infusion in elderly high risk acute myeloid leukemia patients. Blood. (2011) 118:3273–9. 10.1182/blood-2011-01-329508 21791425

[323] Geller MA Cooley S Judson PL Ghebre R Carson LF Argenta PA. A phase II study of allogeneic natural killer cell therapy to treat patients with recurrent ovarian and breast cancer. Cytotherapy. (2011) 13:98–107. 10.3109/14653249.2010.515582 20849361 PMC3760671

[324] Zhu H Yang X Li J Ren Y Zhang T Zhang C. Immune response, safety, and survival and quality of life outcomes for advanced colorectal cancer patients treated with dendritic cell vaccine and cytokine-induced killer cell therapy. Biomed Res Int. (2014) 2014:603871. 10.1155/2014/603871 25136601 PMC4124766

[325] Zhang L Yang X Sun Z Li J Zhu H Li J. Dendritic cell vaccine and cytokine-induced killer cell therapy for the treatment of advanced non-small cell lung cancer. Oncol Lett. (2016) 11:2605–10. 10.3892/ol.2016.4273 27073525 PMC4812113

[326] Poschke I Lövgren T Adamson L Nyström M Andersson E Hansso J. A phase I clinical trial combining dendritic cell vaccination with adoptive T cell transfer in patients with stage IV melanoma. Cancer Immunol Immunother. (2014) 63:1061–71. 10.1007/s00262-014-1575-2 24993563 PMC11028804

[327] Chodon T Comin-Anduix B Chmielowski B Koya RC Wu Z Auerbach M. Adoptive transfer of MART-1 T-cell receptor transgenic lymphocytes and dendritic cell vaccination in patients with metastatic melanoma. Clin Cancer Res. (2014) 20:2457–65. 10.1158/1078-0432.CCR-13-3017 24634374 PMC4070853

[328] Karnoub AE Dash AB Vo AP Sullivan A Brooks MW Bell GW. Mesenchymal stem cells within tumour stroma promote breast cancer metastasis. Nature. (2007) 449:557–63. 10.1038/nature06188 17914389

[329] García-Castro J Alemany R Cascalló M Martínez-Quintanilla J Del Mar Arriero M Lassaletta Á. Treatment of metastatic neuroblastoma with systemic oncolytic virotherapy delivered by autologous mesenchymal stem cells: an exploratory study. Cancer Gene Ther. (2010) 17:476–83. 10.1038/cgt.2010.4 20168350

[330] Melen GJ Franco-Luzón L Ruano D González-Murillo Á Alfranca A Casco F. Influence of carrier cells on the clinical outcome of children with neuroblastoma treated with high dose of oncolytic adenovirus delivered in mesenchymal stem cells. Cancer Lett. (2016) 371:161–70. 10.1016/j.canlet.2015.11.036 26655276

[331] Toda S. Synthetic tissue engineering: programming multicellular self-organization by designing customized cell-cell communication. Biophys Physicobiology. (2020) 17:42. 10.2142/biophysico.BSJ-2020002 33173713 PMC7593132

[332] Bora P Majumdar AS. Adipose tissue-derived stromal vascular fraction in regenerative medicine: a brief review on biology and translation. Stem Cell Res Ther. (2017) 8:145. 10.1186/s13287-017-0598-y 28619097 PMC5472998

[333] You D Jang MJ Kim BH Song G Lee C Suh N. Comparative study of autologous stromal vascular fraction and adipose-derived stem cells for erectile function recovery in a rat model of cavernous nerve injury. Stem Cells Transl Med. (2015) 4:351–8. 10.5966/sctm.2014-0161 25792486 PMC4367505

[334] van Dijk A Naaijkens BA Jurgens WJFM Nalliah K Sairras S van der Pijl RJ. Reduction of infarct size by intravenous injection of uncultured adipose derived stromal cells in a rat model is dependent on the time point of application. Stem Cell Res. (2011) 7:219–29. 10.1016/j.scr.2011.06.003 21907165

[335] Semon JA Zhang X Pandey AC Alandete SM Maness C Zhang S. Administration of murine stromal vascular fraction ameliorates chronic experimental autoimmune encephalomyelitis. Stem Cells Transl Med. (2013) 2:789–96. 10.5966/sctm.2013-0032 23981726 PMC3785263

[336] Ryu DJ Jeon YS Park JS Bae GC Kim J Kim MK. Comparison of bone marrow aspirate concentrate and allogenic human umbilical cord blood derived mesenchymal stem cell implantation on chondral defect of knee: assessment of clinical and magnetic resonance imaging outcomes at 2-year follow-up. Cell Transplant. (2020) 29:0963689720943581. 10.1177/0963689720943581 32713192 PMC7563925

[337] Lee N-H Na S-M Ahn H-W Kang J-K Seon J-K Song E-K. Allogenic human umbilical cord blood-derived mesenchymal stem cells is more effective than bone marrow aspiration concentrate for cartilage regeneration after high tibial osteotomy in medial unicompartmental osteoarthritis of knee. Arthroscopy. (2021) 37:2521–30. 10.1016/j.arthro.2021.02.022 33621649

[338] Eder C Schmidt-Bleek K Geissler S Sass FA Maleitzke T Pumberger M. Mesenchymal stromal cell and bone marrow concentrate therapies for musculoskeletal indications: a concise review of current literature. Mol Biol Rep. (2020) 47:4789–814. 10.1007/s11033-020-05428-0 32451926 PMC7295724

[339] Zhao D Cui D Wang B Tian F Guo L Yang L. Treatment of early stage osteonecrosis of the femoral head with autologous implantation of bone marrow-derived and cultured mesenchymal stem cells. Bone. (2012) 50:325–30. 10.1016/j.bone.2011.11.002 22094904

[340] Chen C Qu Z Yin X Shang C Ao Q Gu Y. Efficacy of umbilical cord-derived mesenchymal stem cell-based therapy for osteonecrosis of the femoral head: A three-year follow-up study. Mol Med Rep. (2016) 14:4209. 10.3892/mmr.2016.5745 27634376 PMC5101965

[341] Tabatabaee RM Saberi S Parvizi J Mortazavi SMJ Farzan M. Combining concentrated autologous bone marrow stem cells injection with core decompression improves outcome for patients with early-stage osteonecrosis of the femoral head: a comparative study. J Arthroplasty. (2015) 30:11–5. 10.1016/j.arth.2015.06.022 26143238

[342] Mao Q Jin H Liao F Xiao L Chen D Tong P. The efficacy of targeted intraarterial delivery of concentrated autologous bone marrow containing mononuclear cells in the treatment of osteonecrosis of the femoral head: a five year follow-up study. Bone. (2013) 57:509–16. 10.1016/j.bone.2013.08.022 23994171 PMC3927161

[343] Wang Q-J Wang H Pan K Li Y-Q Huang L-X Chen S-P. Comparative study on anti-tumor immune response of autologous cytokine-induced killer (CIK) cells, dendritic cells-CIK (DC-CIK), and semi-allogeneic DC-CIK. Chin J Cancer. (2010) 29:641–8. 10.5732/cjc.009.10772 20591215

[344] Saxena M Bhardwaj N. Re-emergence of dendritic cell vaccines for cancer treatment. Trends in cancer. (2018) 4:119–37. 10.1016/j.trecan.2017.12.007 29458962 PMC5823288

[345] Mosińska P Gabryelska A Zasada M Fichna J. Dual functional capability of dendritic cells – cytokine-induced killer cells in improving side effects of colorectal cancer therapy. Front Pharmacol. (2017) 0:126. 10.3389/fphar.2017.00126 28352234 PMC5348514

[346] Koike N Pilon-Thomas S Mulé JJ. Nonmyeloablative chemotherapy followed by T-cell adoptive transfer and dendritic cell-based vaccination results in rejection of established melanoma. J Immunother. (2008) 31:402–12. 10.1097/CJI.0b013e31816cabbb 18391755

[347] Lutz-Nicoladoni C Wallner S Stoitzner P Pircher M Gruber T Wolf AM. Reinforcement of cancer immunotherapy by adoptive transfer of cblb-deficient CD8+ T cells combined with a DC vaccine. Immunol Cell Biol. (2012) 90:130–4. 10.1038/icb.2011.11 21383769

[348] Park M-Y Kim C-H Sohn H-J Oh S-T Kim S-G Kim T-G. The optimal interval for dendritic cell vaccination following adoptive T cell transfer is important for boosting potent anti-tumor immunity. Vaccine. (2007) 25:7322–30. 10.1016/j.vaccine.2007.08.037 17889413

[349] Song S Zhang K You H Wang J Wang Z Yan C. Significant anti-tumour activity of adoptively transferred T cells elicited by intratumoral dendritic cell vaccine injection through enhancing the ratio of CD8(+) T cell/regulatory T cells in tumour. Clin Exp Immunol. (2010) 162:75–83. 10.1111/j.1365-2249.2010.04226.x 20735440 PMC2990932

[350] Tamai H Watanabe S Zheng R Deguchi K Cohen PA Koski GK. Effective treatment of spontaneous metastases derived from a poorly immunogenic murine mammary carcinoma by combined dendritic-tumor hybrid vaccination and adoptive transfer of sensitized T cells. Clin Immunol. (2008) 127:66–77. 10.1016/j.clim.2007.12.001 18262845

[351] Johnson MB March AR Morsut L. Engineering multicellular systems: using synthetic biology to control tissue self-organization. Curr Opin Biomed Eng. (2017) 4:163–73. 10.1016/j.cobme.2017.10.008 29308442 PMC5749256

[352] Bracci L Fragale A Gabriele L Moschella F. Towards a systems immunology approach to unravel responses to cancer immunotherapy. Front Immunol. (2020) 0:2748. 10.3389/fimmu.2020.582744 33193392 PMC7649803

[353] Davis MM Tato CM Furman D. Systems immunology: just getting started. Nat Immunol. (2017) 18:725–32. 10.1038/ni.3768 28632713 PMC5790187

[354] Han F Lu P. Future challenges and perspectives for stem cell therapy of neurodegenerative diseases. Adv Exp Med Biol. (2020) 1266:141–5. 10.1007/978-981-15-4370-8_10 33105500

[355] Abreu TR Fonseca NA Gonçalves N Moreira JN. Current challenges and emerging opportunities of CAR-T cell therapies. J Control Release. (2020) 319:246–61. 10.1016/j.jconrel.2019.12.047 31899268

[356] Baruch EN Berg AL Besser MJ Schachter J Markel G. Adoptive T cell therapy: an overview of obstacles and opportunities. Cancer. (2017) 123:2154–62. 10.1002/cncr.30491 28543698

[357] DVC Stem. The Cost of Stem Cell Therapy in 2021. (2021). Available online at: https://www.dvcstem.com/post/stem-cell-therapy-cost-2020 (accessed July 25, 2021).

[358] NIKHIL VERMA MD. Regenerative Medicine Injection Pricing. (2017). Available online at: https://www.sportssurgerychicago.com/2017/10/05/regenerative-medicine-injection-pricing/ (accessed July 25, 2021).

